# Weight loss moderately affects the mixed meal challenge response of the plasma metabolome and transcriptome of peripheral blood mononuclear cells in abdominally obese subjects

**DOI:** 10.1007/s11306-018-1328-x

**Published:** 2018-03-05

**Authors:** Parastoo Fazelzadeh, Roland W. J. Hangelbroek, Peter J. Joris, Casper G. Schalkwijk, Diederik Esser, Lydia Afman, Thomas Hankemeier, Doris M. Jacobs, Velitchka V. Mihaleva, Sander Kersten, John van Duynhoven, Mark V. Boekschoten

**Affiliations:** 1 Nutrition, Metabolism and Genomics Group, Division of Human Nutrition, Wageningen University, Wageningen, The Netherlands; 2 Department of Human Biology, NUTRIM School of Nutrition and Translational Research in Metabolism, Maastricht University, Maastricht, The Netherlands; 3 Department of Internal Medicine, CARIM School for Cardiovascular Diseases, Maastricht University Medical Center, Maastricht, The Netherlands; 4 Laboratory of Biophysics, Wageningen University, Stippeneng 4, 6708WE, Wageningen, The Netherlands; 5 Top Institute Food and Nutrition, Wageningen, The Netherlands; 6 Netherlands Metabolomics Centre, Leiden, The Netherlands; 7 Unilever R&D, Vlaardingen, The Netherlands

**Keywords:** Metabolic health, Mixed-meal challenge, Phenotype shift

## Abstract

**Introduction:**

The response to dietary challenges has been proposed as a more accurate measure of metabolic health than static measurements performed in the fasted state. This has prompted many groups to explore the potential of dietary challenge tests for assessment of diet and lifestyle induced shifts in metabolic phenotype.

**Objectives:**

We examined whether the response to a mixed-meal challenge could provide a readout for a weight loss (WL)-induced phenotype shift in abdominally obese male subjects. The underlying assumption of a mixed meal challenge is that it triggers all aspects of phenotypic flexibility and provokes a more prolonged insulin response, possibly allowing for better differentiation between individuals.

**Methods:**

Abdominally obese men (n = 29, BMI = 30.3 ± 2.4 kg/m^2^) received a mixed-meal challenge prior to and after an 8-week WL or no-WL control intervention. Lean subjects (n = 15, BMI = 23.0 ± 2.0 kg/m^2^) only received the mixed meal challenge at baseline to have a benchmark for WL-induced phenotype shifts.

**Results:**

Levels of several plasma metabolites were significantly different between lean and abdominally obese at baseline as well as during postprandial metabolic responses. Genes related to oxidative phosphorylation in peripheral blood mononuclear cells (PBMCs) were expressed at higher levels in abdominally obese subjects as compared to lean subjects at fasting, which was partially reverted after WL. The impact of WL on the postprandial response was modest, both at the metabolic and gene expression level in PBMCs.

**Conclusion:**

We conclude that mixed-meal challenges are not necessarily superior to measurements in the fasted state to assess metabolic health. Furthermore, the mechanisms accounting for the observed differences between lean and abdominally obese in the fasted state are different from those underlying the dissimilarity observed during the postprandial response.

**Electronic supplementary material:**

The online version of this article (10.1007/s11306-018-1328-x) contains supplementary material, which is available to authorized users.

## Introduction

Healthy individuals are able to respond to external stimuli by keeping biological parameters within narrow homeostatic bandwidths. As a consequence, current approaches focusing on disease risk biomarkers mostly lack the sensitivity to detect the effects of lifestyle and dietary interventions aimed at improving or sustaining health. Hence, there is a growing awareness that ‘health’ should not just be defined as ‘the absence of disease’, but is more accurately described as ‘resilience of homeostatic control’, i.e., the ability to cope with daily challenges (Huber et al. 2011) without drifting out of the regulated homeostatic/allostatic zone (Ommen et al. 2009). The concept of the human body as an orchestrated machine that continuously adapts to a changing environment has been embraced by the nutritional field and coined as ‘phenotypic flexibility’ (Ommen et al. 2014). The earlier introduced concept of ‘metabolic flexibility’ departs from a narrower definition of phenotypic flexibility and specifically refers to the ability of organs to change fuel use depending on availability (Corpeleijn et al. 2009). The capacity to switch from carbohydrate to fat oxidation and vice versa depending on their supply and demand is crucial for optimal metabolic homeostasis, and thus an important aspect of phenotypic flexibility. In both concepts, the resilience capacity can be tested by the assessment of the stress response to short-term perturbations.

Specific dietary challenge tests have been described, each probing the resilience of different metabolic regulatory processes (Ommen et al. 2009). The best known challenge test is the oral glucose tolerance test (OGTT), which specifically probes the resilience of glucose metabolism (Ho et al. 2013). The oral lipid and protein tolerance tests (OLTT and OPTT, respectively) probe the resilience of other, and partially overlapping metabolic regulatory processes (Stroeve et al. 2015; Krug et al. 2012; Wopereis et al. 2013). The use of a mixed-meal challenge, comprising protein, glucose and lipids, has been proposed to target multiple organs and more broadly encompass phenotypic flexibility (Stroeve et al. 2015; Wopereis et al. 2017). By challenging metabolic regulatory processes by means of a dietary challenge, dynamic changes in nutrient metabolism might be uncovered, allowing better exploration of the individual capacity to cope with metabolic stressors (Krug et al. 2012; Broek et al. 2017).

Next to metabolite profiling, which has already been shown to be useful in revealing the complex changes upon dietary challenges (Ommen et al. 2014; Stroeve et al. 2015), gene expression of peripheral blood mononuclear cells (PBMCs) has been proposed as a readout of biological processes such as inflammation, metabolism (Afman et al. 2014), oxidative stress and inflammatory status (Crujeiras et al. 2008; Sheu et al. 2008). PBMCs can be easily collected in adequate quantities for transcriptomics studies, and it has been shown that nutritional components can modulate pro- and anti-inflammatory mechanisms (Afman et al. 2014; Esser et al. 2015). A previous study indicated that long-term consumption of a Mediterranean diet (high content of unsaturated fats and polyphenols) reduces metabolic stress and oxidative phosphorylation activity in PBMCs obtained from overweight men and women (Dijk et al. 2012). Furthermore, it has also been shown that diet-induced weight loss (WL) modulates immune-inflammatory and antioxidant responses and mRNA expression in PBMCs (Mello et al. 2012).

We examined whether a mixed-meal challenge response could provide a sensitive readout for a shift in phenotypical flexibility upon WL in abdominally obese male subjects. By combining metabolite profiling in plasma and whole genome gene expression in PBMCs, we aimed at comprehensively describing the changes in the metabolome and gene expression underlying phenotypic flexibility. We were in particular interested whether the effect of WL on the postprandial response would provide a more sensitive readout than the observation in the fasting state. In order to have a benchmark for this comparison, we first compared fasting baseline and postprandial response between lean and abdominally obese subjects. Subsequently, we assessed the effect of dietary WL accompanied by improvements in vascular function, lipid profile, and insulin sensitivity on the fasting baseline and postprandial response in abdominally obese men.

## Materials and methods

### Subject characteristics

Fifteen lean men with a waist circumference below 94 cm (BMI = 23.0 ± 2.0 kg/m^2^) and 29 abdominally obese men with a waist circumference between 102 and 110 cm participated in the study (BMI = 30.3 ± 2.4 kg/m^2^). Baseline characteristics of subjects of which microarrays and metabolomics were performed are displayed in Table 1. All volunteers were apparently healthy and did not receive proton pump inhibitors, anti-hypertensive medication or drugs known to affect lipid or glucose metabolism. All participants gave written, informed consent before entering the study. The study was approved by the Medical Ethics Committee of Maastricht University Medical Center and registered at clinicaltrials.gov as NCT01675401 (Joris et al. 2016).

**Table 1:** Characteristics of lean subjects and abdominally obese subjects before and after weight loss or control interventions

	Lean	WL(D1)	WL(D2)	CTRL(D1)	CTRL(D2)
Number	15	14	14	15	15
Age (years)	47.4 ± 17	44 ± 15		44.8 ± 14.0	
Weight (kg)	73.0 ± 7.5	96.6 ± 8.6	87.3 ± 8.6*	98.9 ± 9.9	98.1 ± 9.5
Height (m)	1.77 ± 0.07	1.79 ± 0.05		1.80 ± 0.07	
BMI (kg/m^2^)	23.0 ± 2.0	30.0 ± 1.8	26.9 ± 1.6*	30.7 ± 2.9	30.4 ± 2.8
Waist circumference (cm)	85.4 ± 6.7	106.8 ± 3.6	95.7 ± 4.5*	106.8 ± 3.9	106.2 ± 3.8
Hip circumference (cm)	95.9 ± 4.1	108.4 ± 4.9	102.7 ± 4.5*	109.5 ± 7.0	109.3 ± 7.8
LBM (kg)	54.2 ± 6.7	67.1 ± 6.9	61.5 ± 8.9	69.2 ± 10.4	69.8 ± 10.7
Fat mass (%)	19.0 ± 6.5	29.5 ± 4.4	25.8 ± 5.0	29.8 ± 4.3	28.3 ± 4.1
Glucose (mmol/L)	5.1 ± 2.9	5.3 ± 0.47	5.0 ± 0.33	5.3 ± 0.48	5.3 ± 0.37
Insulin (µU/mL)	7.0 ± 1.7	12.5 ± 5.5	7.8 ± 3.4*	12.0 ± 6.5	12.1 ± 5.2

Data are presented as mean ± SD

**Fig. 1:**
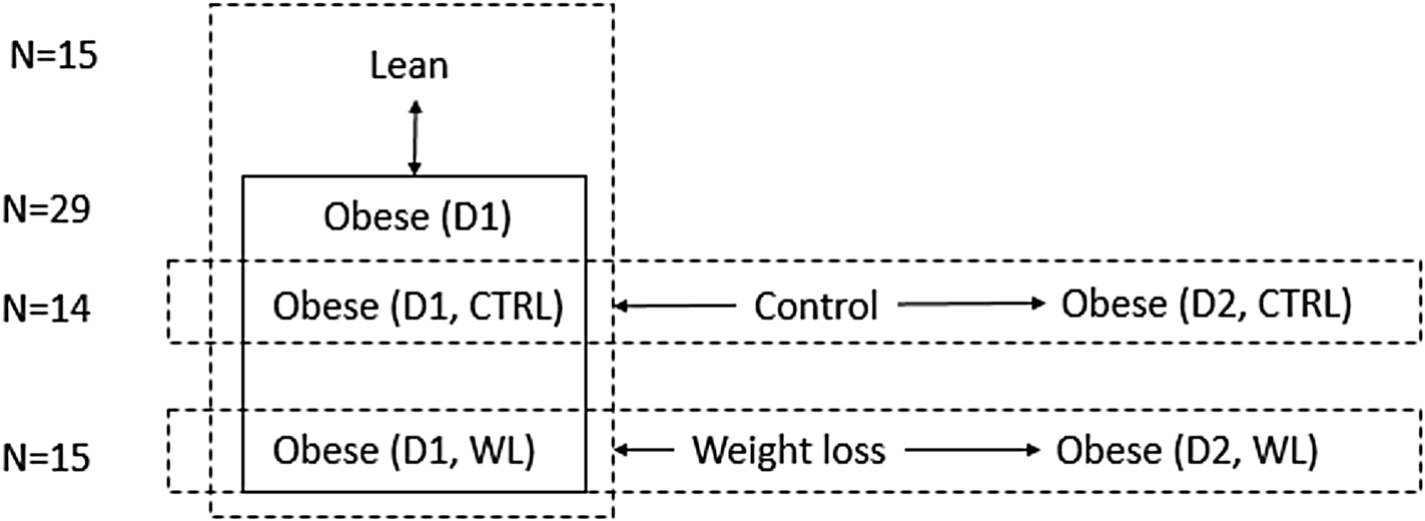
Schematic overview of the study design. *D1, D2* before and after intervention, respectively, *WL* weight loss intervention group, *CTRL* control group. The arrows indicate the phenotype comparisons made in this study (lean vs. abdominally obese, before and after interventions)

*WL* weight loss intervention, *CTRL* control group, *D1, D2* days before and after intervention (see also Fig. 1), *BMI* body mass index, *LBM* lean body mass

*A significant effect of weight loss (*P* < 0.05)

### Study design

Full details of the study have been published before (Joris et al. 2016). In brief, abdominally obese men received a mixed-meal challenge prior to and after an 8-week WL or no-WL control intervention. Lean subjects were included as a reference group and only received the mixed meal challenge at baseline (Fig. 1). Power calculations were based on an α of 0.05, and a within-subject and between-subject variability of 2.82 percentage points in the primary outcome variable FMD (Joris et al. 2016). Prior to the intervention (D1), all subjects underwent a mixed-meal challenge. The standardized mixed meal consisted of two muffins and 300 mL 0% fat milk, which provided 4.6 MJ (4598 kJ or 1100 kcal): 56.6 g fat, 26.5 g protein and 121 g carbohydrate. Blood samples were collected at fasting and immediately after ingestion of the challenge over 4 h at regular intervals (0, 30, 60, 120, 180, 240 min). Subjects assigned to diet-induced WL (Fig. 1) program consumed a commercially available very low energy diet (Modifast; Nutrition et Santé, Benelux, Breda, The Netherlands) for 4–5 weeks providing 2.1 MJ/day. Once the waist circumference was below 102 cm (the NCEP ATP II cut-off value) within this period, subjects consumed a mixed-solid energy-restricted diet up to 4.2 MJ/day with a recommended composition for the following 1–2 weeks. Then, subjects consumed a diet matching their energy requirements to maintain their newly achieved body weights (weight-stable conditions) for at least 2 weeks. Subjects underwent the same mixed-meal challenge again after the WL intervention (D2). Blood samples were collected at the same time intervals as on D1. Subjects assigned to the control (CTRL, Fig. 1) intervention underwent the same tests, but maintained their habitual diet, physical activity levels and alcohol consumption during 8 weeks. The period between the first and second measurements was the same for the WL intervention and control (CTRL) groups. More details on the composition of low energy diet and mixed meal challenge can be found elsewhere (Joris et al. 2016).

### Sample collection

On the day before blood sampling, subjects were asked not to perform any strenuous physical exercise or to consume alcohol. On the morning of blood sampling—after a 12 h overnight fast (from 8 p.m.) subjects were only allowed to drink a glass of water in the morning. Subjects were also asked not to consume high-fat foods on the day prior to the test days and to come to the test centre by public transport or car to standardize measurements as much as possible.

An intravenous cannula was inserted, and blood samples were taken at fasting and after mixed meal consumption both before and after the WL intervention at six time points [fasting (T0) and 30, 60,120,180 and 240 min in the postprandial state]. Metabolic profiling was performed on all time points and transcriptomics analysis of PBMCs only at fasting (T0) and after 4 h in the postprandial state (T4).

### PBMC and RNA isolation

PBMCs were isolated before and 4 h after mixed meal challenge by using BD Vacutainer Cell Preparation Tubes. RNA was isolated (RNeasy Micro kit, Qiagen, Venlo, The Netherlands), quantified (Nanodrop ND 1000, Nanodrop Technologies, Wilmington, DE, USA) and integrity was checked by an Agilent 2100 Bioanalyser with RNA 6000 microchips (Agilent Technologies, South Queensferry, UK). Samples were included for microarray analysis if RNA integrity number was > 7.

### Microarray processing

PBMC samples from 15 lean and 29 abdominally obese subjects yielded enough RNA of sufficient quality at all collection points to perform microarray analysis. Microarray analysis was performed for each individual at fasting (T0) and 4 h (T4) in the postprandial state. The 4 h timepoint was chosen for transcriptomics analysis to allow for comparison to previous studies (Esser et al. 2015). Total RNA was labelled using a one-cycle cDNA labelling kit (MessageAmp™ II-Biotin Enhanced Kit; Ambion, Inc., Nieuwerkerk a/d IJssel, Netherlands) and hybridized to GeneChip® Human Gene 1.1 ST Array targeting 19 738 unique gene identifiers (Affymetrix, Inc., Santa Clara, CA, USA). Sample labelling, hybridization to chips, and image scanning were performed according to the manufacturers’ instructions.

### Microarray analysis

Microarray signals were normalized using the robust multichip average algorithm. Data was filtered using universal expression codes filtering with a 50% cut-off, corresponding to a 50% likelihood that a gene is expressed (Piccolo et al. 2012). Significant differences of individual genes were tested using the limma R library (Smyth 2005). At fasting, the expression of genes between groups was defined as different when *P* was < 0.05 in an unpaired t-test with empirical Bayes correction. Gene expression was defined as postprandial changed between T0 and T4 h when *P* < 0.05 in a paired t-test with empirical Bayesian correction. Data were further analysed with gene set enrichment analysis (GSEA) using pre-ranked lists based on the t-statistic (Subramanian et al. 2005). Gene sets with a false discovery rate (FDR Q < 0.2) were defined as significantly regulated. Pathway analyses were performed using ingenuity pathway analysis (Qiagen, Redwood City, http://www.qiagen.com/ingenuity) on filtered dataset and filtered genes were used as background. Plots were made using the R libraries ggplot2 and gplots (Wickham 2009; Warnes et al. 2009).

### Plasma metabolic profiling

Amino acids and biogenic amines were derivatized (Acc-Tag) in 5 µL aliquots of plasma and analyzed using an Acquity UPLC system equipped with autosampler (Waters, Etten-Leur, The Netherlands) and coupled to a Xevo Tandem quadrupole mass spectrometer (Waters) operated using QuanLynx data acquisition software (version 4.1; Waters). An Accq-Tag Ultra column (Waters) was used. The Xevo TQ was used in the positive-ion electrospray mode and all analytes were monitored in multiple reaction monitoring (MRM) using nominal mass resolution. Acquired data were evaluated using TargetLynx software (Waters), by integration of assigned MRM peaks and normalization using proper internal authentic standards (Noga et al. 2012). For analysis of amino acids, their ^13^C–^15^N labeled analogs were used and for other amines the closest-eluting internal standard was employed.

Acylcarnitines, trimethylamine-N-oxide, choline, betaine, deoxycarnitine and carnitine were analyzed in 5 µL plasma, spiked with an internal authentic standard, using a UPLC–MS/MS. Also here an Accq-Tag Ultra column was used. The Xevo TQ was used in the positive-ion electrospray mode and all analytes were monitored in MRM using nominal mass resolution. In-house developed algorithms (Kloet et al. 2009) were applied using the pooled QC samples to compensate for shifts in the sensitivity of the mass spectrometer over the batch. Organic acids were measured by GC–MS using 50 µL of plasma sample prepared using a two-step derivatization procedure with subsequent oximation using methoxyamine hydrochloride (MeOX) and silylation using *N*-methyl-*N*-(trimethylsilyl)trifluoroacetamide (MSTFA). Samples were measured on an Agilent GC (7890A) coupled to Agilent Quadrupole-MS with EI source (Agilent MSD 5975C). Separation was performed using a HP-5MS column (30 m × 0.25 m × 0.25 µm; Agilent). The raw data were pre-processed using Agilent MassHunter Quantitative Analysis software for GC–MS (Agilent, Version B.04.00), and quantitation of metabolite response was calculated as the peak area ratios of the target analyte to the respective internal authentic standard. Oxylipins were analyzed as previously described (Strassburg et al. 2012). In short, compound extraction was performed with SPE using a hydrophilic–lipophilic balance and samples were analyzed by LC using a C18 column coupled to ESI on a triple quadrupole mass spectrometer, where oxylipins were detected in negative ion mode using dynamic SRM, while chromatographic separation was achieved with a C18 column. Peak areas of target metabolites were corrected by appropriate internal authentic standards and calculated response ratios were used throughout the analysis. Quality controls were run every 10 samples and before and after calibration samples, amounting to a total of 62 QC samples per platform. In-house developed algorithms were applied using the pooled QC samples to compensate for shifts in the sensitivity of the mass spectrometer over the batch. Serum metabolite concentrations determined by NMR were measured as described by Mihaleva et al. (2014). Briefly, high resolution ^1^H NMR spectra were acquired on ultrafiltrated serum samples at 300 K using a Bruker Avance III 600 MHz spectrometer. Automated quantum mechanical line shape fitting of the ^1^H NMR spectra was performed using PERCH NMR software to obtain absolute concentrations of 44 metabolites. The median in the RSDs of metabolite concentrations in the QC samples measured for all platforms was 8%. All metabolites reported in this study were identified at MSI level 1.

### Metabolomics statistical analysis

We used Spearman rank correlations to correlate plasma metabolite concentrations and phenotypical parameters (LBM, HOMA). These correlations were also determined for nadir and Δ-nadir acylcarnitine values (Ramos-Roman et al. 2012). Nadir acylcarnitine values were defined as the lowest value achieved during the 4 h after the meal. Δ-Nadir was calculated as difference between nadir and T0 values. The postprandial response was considered as the incremental area under the curve (iAUC) (Carstensen et al. 2003). For the iAUC calculation, the values at fasting (T0) were subtracted from the total AUC. Comparison between abdominally obese and lean group was performed using analysis of variance. To evaluate the intervention effect on fasting metabolites (T0) and the intervention effect on postprandial response (iAUC) in abdominally obese subjects, statistical analysis was performed using linear mixed models. A *P* < 0.05 was considered to be statistically significant. Local FDR (lFDR) was used to correct for multiple testing (Strimmer 2008; Box et al. 1994). All calculations were performed using R (version 3.2.1).

## Results

### Metabolic differences between lean and abdominally obese subjects

**Fig. 2:**
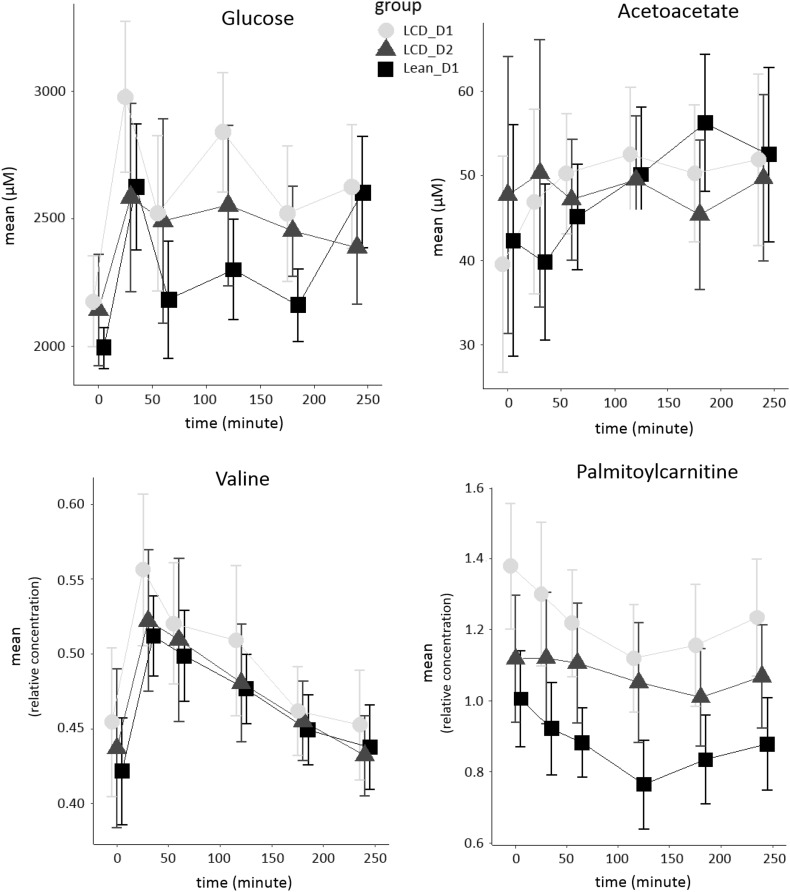
Postprandial mixed meal response curves of glucose, acetoacetate, valine and palmitoylcarnitine as representative metabolites from glycolysis, ketogenesis, amino acid metabolism and lipolysis, respectively. Mean curves are presented for lean and abdominally obese subjects, the latter before and after weight loss intervention (see Fig. 1). The bars represent variation within these groups. The metabolite levels for glucose and acetoacetate are presented as absolute concentrations, for valine and palmitoylcarnitine relative concentrations are presented

In order to have a benchmark for the comparison of abdominally obese subjects before and after WL, we first considered the difference between lean and abdominally obese subjects (Fig. 1). In the fasted state, 19 plasma metabolites were significantly different between abdominally obese subjects and lean subjects (Table 2), including branched chain and other amino acids, a fatty acid derived acylcarnitine (FAAC), and metabolites related to the TCA cycle. For a number of oxylipins we observed significantly lower plasma levels in the abdominally obese subjects compared to the lean subjects. Upon the mixed meal challenge, four types of postprandial metabolic responses could be recognized, i.e., metabolite changes related to glucose metabolism, amino acid metabolism, lipid metabolism and ketogenesis (Shaham et al. 2008). Figure 2 presents typical postprandial curves for these response types. Both lean and abdominally obese subjects followed similar responses over time. Metabolites for which the longitudinal response, expressed as iAUC, significantly (P < 0.05) differed between abdominally obese and lean subjects are presented in Table 2. The eight metabolites that were different between lean and abdominally obese included a number of amino acids, but not branched chain amino acids. Interestingly, 2-hydroxyisovalerate, a catabolite of branched chain amino acids was also significantly different.

**Table 2:** Significant (P < 0.05, lFDR < 0.2) differences between fasting metabolite concentrations (FC) and postprandial mixed meal response (iAUC) in plasma between lean and abdominally obese subjects

	*P*(fasting)	FC (abdominally obese T0/lean T0)	*P*(iAUC)
Acylcarnitines			
Palmitoylcarnitine	< 0.01	1.25	
Amino acids and related metabolites			
Serine	< 0.01	0.86	
Beta alanine	0.02	1.07	
Asparagine	0.02	0.9	
Creatinine	< 0.01	1.21	
Isoleucine	< 0.01	1.24	
Alanine	< 0.01	1.18	< 0.01
Tyrosine	< 0.01	1.2	
Valine	0.01	1.13	
Keto-leucine	0.01	1.13	
Carnitine	0.02	1.07	
Leucine	0.03	1.11	
Proline			< 0.01
Methylmalonic acid			< 0.01
Threonine			0.01
Histidine			0.01
Methionine			0.01
Phosphocholine			0.01
2-Hydroxyisovalerate			0.01
Oxylipins			
5-HETE	< 0.01	0.61	
12–13-EpOME	0.01	0.65	
9-HODE	0.02	0.71	
9-HOTrE	0.02	0.68	
TCA cycle and related metabolites			
Glyceric acid	< 0.01	0.95	
Beta glucose	0.02	1.09	

Differences in response were calculated on the basis of iAUC

*FC* fold change, *iAUC* incremental area under the curve

In the fasted state, 1862 genes were differently expressed (*P* < 0.05) in PBMCs between lean and abdominally obese subjects. The mixed meal challenge changed the expression of 1305 genes in abdominally obese and 1707 genes in lean subjects. The response to the meal challenge of 1537 genes differed between lean and abdominally obese (Δ abdominally obese T4–T0 vs. Δ lean T4–T0). A total of 359 genes were both differentially expressed between abdominally obese and lean after fasting and differed between abdominally obese and lean subjects in response to the mixed meal challenge (Fig. S1). In the fasting samples, GSEA identified gene sets that were enriched in abdominally obese compared to lean subjects (FDR < 0.2), which mainly belonged to oxidative phosphorylation and the electron transport chain. Gene sets involved in immune regulation were enriched in lean compared to abdominally obese subjects (Supplementary Table S1). In the postprandial state, genes related to immune pathways and glucose metabolism were enriched in abdominally obese as compared to lean (Δ abdominally obese T4–T0 vs. Δ lean T4–T0, Supplementary Table S2). In total, in the fasting state nine gene sets were positively enriched and nine gene sets were negatively enriched in abdominally obese compared to lean subjects (FDR < 0.2). In the postprandial state, 114 gene sets were upregulated in abdominally obese subjects compared to lean subjects, while 15 gene sets were downregulated.

### Impact of weight loss on fasting metabolism in abdominally obese subjects

All subjects who received the low-calorie diet significantly lost weight (*P* < 0.05) (Table 1). To establish whether WL has any effect on the fasting levels of metabolites in abdominally obese subjects, Δ values were calculated per metabolite per subject (T0 after –T0 before). Table 3 shows the three metabolites that were different before and after the WL intervention in abdominally obese subjects in the fasted state. We also assessed the connection between WL and ratios between acetylcarnitine (C_2_) and longer chain (C_n_) acylcarnitines. These (C_2_/C_n_) acylcarnitine ratios have previously been suggested as read-outs for lipid β-oxidation and indeed significantly correlated with HOMA (Supplementary Material, Table S3). Although these correlations suggest that acylcarnitine ratios partly reflect insulin resistance, we could not find any significant effect of WL on these acylcarnitine ratios, whereas we did observe an improvement in the HOMA index (Joris et al. 2016).

**Table 3:** Effect of weight loss (P < 0.05, lFDR < 0.2) on fasted state (T0, FC) metabolite concentrations and postprandial mixed meal challenge response (iAUC) in plasma of abdominally obese subjects

Metabolites	*P*	FC(WL)	*P*(iAUC)
Amino acids and related metabolites			
Glycine	< 0.01	1.18	
Creatinine	< 0.01	0.88	
Glutamine			< 0.01
Histidine			< 0.01
Creatine			< 0.01
Pyroglutamic acid			< 0.01
Glutamic acid	< 0.01	0.66	
Oxylipins			
TXB2			< 0.01
PGE2			< 0.01
12S.HHTrE			< 0.01
5.HETE			< 0.01
11.HETE			0.02
TCA cycle and related metabolites			
Glucose			0.01
Acylcarnitines			
Choline			0.01

Differences in postprandial response were determined on the basis of iAUC

*FC* fold change (T0, after weight loss/T0, before weight loss), *iAUC* incremental area under the curve significant at *P* < 0.05

**Fig. 3:**
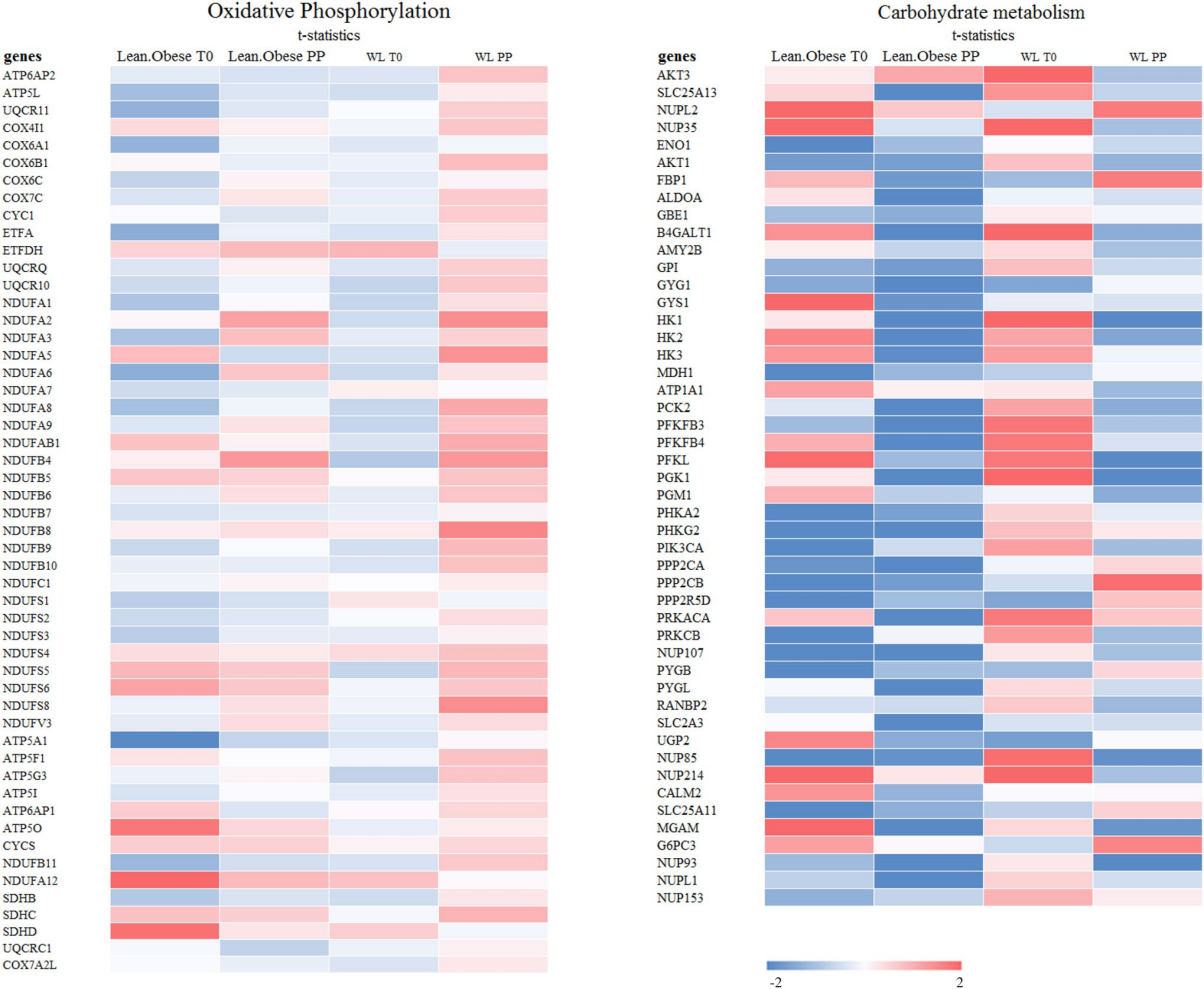
Overview of lean-abdominally obese differences in expression of genes related to (left) oxidative phosphorylation and (right) carbohydrate metabolism in PBMCs at fasting (T0) and during postprandial response (∆ lean vs. ∆ abdominally obese postprandial) to a mixed meal challenge. Also shown are effects of WL on gene expression in PBMCs of abdominally obese subjects at baseline (abdominally obese after WL vs. abdominally obese before WL) and during postprandial phase (∆ abdominally obese after WL vs. ∆ abdominally obese before WL). The heatmap is based on moderated t-statistics for each comparison, which has the same interpretation as an ordinary t-statistic except that the standard errors have been moderated across genes using a simple Bayesian model

We next determined the effect of WL on gene expression in PBMCs in the fasted state. WL changed the expression of 835 genes in the abdominally obese subjects (T0 after WL vs. T0 before WL, Fig. S2). GSEA identified a number of differently enriched (FDR < 0.04) pathways, including respiratory electron transport, and oxidative phosphorylation, which were negatively enriched after WL in abdominally obese subjects. Gene sets related to immune regulation and insulin signalling were positively enriched after WL (Supplementary Table S4). After WL, genes in oxidative phosphorylation and to a lesser extent in carbohydrate metabolism showed expression levels that were closer to expression levels in lean subjects (Fig. 3).

### Impact of weight loss on postprandial metabolic response in abdominally obese subjects

The WL intervention had a subtle effect on the postprandial metabolic response in plasma, of which several examples are presented in Fig. 2. For 11 metabolites the postprandial response as expressed as iAUC was significantly different before and after WL intervention. For a major part these metabolites consisted of oxylipins derived from enzymatic oxidation of arachidonic acid (Table 3). We also observed shifts in the response to the mixed-meal challenge after the control intervention (Supplementary Table S5).

To identify differences in PBMC gene expression in response to the mixed-meal challenge before and after WL, differences were calculated as ∆ values in both groups (T4–T0, D2 vs. T4–T0, D1). We identified 384 genes that showed a different postprandial response after the WL intervention as compared to the response before WL. Furthermore, we identified 226 genes that showed a different postprandial response after the control intervention (Fig. S2). GSEA showed that oxidative phosphorylation and the electron transport chain were the main affected pathways. Before the intervention oxidative phosphorylation and the electron transport chain were downregulated upon the mixed-meal challenge, whereas after the WL intervention oxidative phosphorylation and the electron transport chain were upregulated (Fig. 3). In contrast, gene sets related to the immune system and carbohydrate metabolism showed the opposite pattern with down-regulation upon a mixed-meal challenge before the intervention and a dampened response or even up-regulation upon a mixed-meal challenge after the intervention (Fig. 3; Supplementary Table S6).

In short, GSEA showed that at fasting, 161 gene sets were upregulated and 120 gene sets were down regulated (FDR < 0.2) in the WL abdominally obese group (Supplementary Table S4), while in the abdominally obese control group 219 gene sets were upregulated and 3 gene sets were downregulated (data not shown). GSEA showed that upon mixed meal challenge, 6 gene sets were upregulated and 95 gene sets were down regulated (FDR < 0.2) in WL abdominally obese group (Supplementary Table S6), while in CTRL abdominally obese group only 45 gene sets were downregulated (data not shown).

## Discussion

In this study, we examined whether a standardized mixed-meal challenge could reveal a WL-induced shift from the abdominally obese phenotype towards a lean one. We were particularly interested in finding out whether measurement of the response to a dietary challenge would reveal additional changes in metabolic phenotype compared to measurements in the fasting state.

### Metabolic differences between lean and abdominally obese

The observed differences in the fasting levels of BCAAs reflect differences in metabolic homeostasis between lean and abdominally obese and are in line with previous observations (Newgard et al. 2009). This indicates that the study was adequately powered to detect metabolic differences at baseline. The shift in fasting levels of these metabolites can be explained by their positive correlation with insulin resistance, which was significantly different for the lean and abdominally obese subjects (Newgard et al. 2009; Newgard 2012). We could however not confirm previously observed differences in metabolites related to the TCA cycle between the abdominally obese and lean subjects (Newgard et al. 2009). For a range of oxylipins significantly lower fasting plasma levels were observed in abdominally obese subjects relative to the lean group, which we attribute to a decrease in enzymatic lipid oxidation. Recently, ratios between C_2_/C_n_ acylcarnitines were brought forward as putative read-outs for β-oxidation rate (Krug et al. 2012). Although these ratios correlated with HOMA (Supplementary Table S3), we could not find a difference of C_2_/C_n_ acylcarnitine ratios between the lean and abdominally obese groups, even though insulin sensitivity differed between the two groups. This suggests that while these ratios are correlated with insulin sensitivity, they do not provide an increased sensitivity compared to classical markers.

The lean and abdominally obese subjects also differed in their postprandial mixed meal response. The individual variation, however, was rather large (compared to analytical variation), as shown in Fig. 2. An elaborate interpretation of the differences in response between lean and abdominally obese is given in the Supplementary Material. The strong inter-individual variation in response, the confounding of the metabolic response with dietary influx amino acids, and our conservative statistical testing criteria did not allow for integrated metabolic pathway analysis due to the limited number of affected metabolites. We could however reproduce previously observed differences in the postprandial responses of amino acids between lean and abdominally obese (Badoud et al. 2015). We could not reproduce earlier observations showing differences in the postprandial responses of free fatty acids between lean and abdominally obese. We attributed this to dietary intake of fatty acids during the postprandial phase. We also did not observe differences in postprandial response of FAACs and hence could not confirm earlier observations that lean and abdominally obese differ in β-oxidation of fatty acids (Badoud et al. 2015). Also no differences in postprandial responses of BCAAs between lean and abdominally obese could be discerned, very likely also due to direct dietary intake of amino acids. We did however observe a striking difference for a BCAA transamination product, i.e., 2-hydroxyisovalerate (*P* < 0.05). This metabolite requires the branched chain amino transferase and keto acid dehydrogenase (BCKDH) complex for further catabolism to propionic acid. The function of the BCKDH complex is known to be compromised in insulin resistant subjects, leading to increased fasting levels of BCAA (Badoud et al. 2015). Compromised BCKDH function should also increase postprandial accumulation of other upstream metabolites such as 2-hydroxyisovalerate. This is indeed what was observed in the abdominally obese relative to lean subjects (Podebrad et al. 1999). We also observed difference for a range of postprandial oxylipin responses after a mixed meal challenge in lean and abdominally obese subjects. A recent study however did not show differences in postprandial response of oxylipins between lean and abdominally obese subjects upon high fat challenges (Strassburg et al. 2014). This indicates that the postprandial response of oxylipins is not only determined by the lean-abdominally obese phenotype but also depends on the composition of the meal challenge.

Although the lean and abdominally obese showed several distinct differences in the postprandial metabolic response, these differences were less pronounced when compared to a previous study using an OGTT challenge (Geidenstam et al. 2014). That study showed blunted responses of BCAAs and FFAs, in contrast to our study. We attribute this to dietary intake of these metabolites since our mixed meal challenge was quite rich in protein and fatty acids. This likely shrouds observation of differences in postprandial response due to phenotypical differences.

Gene sets related to oxidative phosphorylation were higher expressed in abdominally obese in comparison to lean subjects at baseline in the fasting state. This is consistent with a previous study that found that dietary background can affect PBMC gene expression. Consumption of monounsaturated fatty acids and a Mediterranean diet compared with a saturated fatty acid diet decreased the expression of genes involved in oxidative phosphorylation in abdominally overweight males and females (Dijk et al. 2012). Differences were also found in gene sets related to the immune system. These gene sets were lower expressed in abdominally obese when compared to lean subjects. However, these effects were relatively small, yet consistent with a previous study that also showed rather small differences in a number of immune related gene sets that showed lower expression in abdominally obese subjects when compared to lean subjects (Esser et al. 2015).

In contrast to the fasting state, we observed that in the postprandial state expression of genes related to immune pathways was increased in abdominally obese relative to lean subjects. In addition, we observed larger changes in expression of gene sets involved in carbohydrate metabolism in the abdominally obese, which could be due to differences in insulin sensitivity and the activity of energy metabolism pathways. The observed differences in PBMC gene expression between abdominally obese and lean subjects at baseline and after the mixed meal challenge point towards a difference in haemostasis and immune function between the two groups.

Based on the number of genes differently expressed between lean and abdominally obese we can conclude that our mixed meal challenge was not able to magnify differences in transcriptional response in PBMC between lean and abdominally obese when compared to the fasting state (fasting: 1862 genes and postprandial: 1537 genes). This stands in contrast to a study by Esser et al. demonstrating that an OLTT challenge with 95 g of fat high in SFA or MUFA increased the number of genes significantly differentially expressed between lean and abdominally obese subjects. This study also showed a higher number of changed genes in abdominally obese relative to lean subjects when comparing the MUFA and the SFA challenges. The authors hypothesised that a MUFA challenge is more potent in inducing transcriptional differences between lean and abdominally obese in PBMCs than a SFA challenge. Our mixed meal challenge was higher in carbohydrate and protein and lower in fat content. These fats were also predominantly saturated, which may explain the weaker gene expression response to our mixed meal challenge. This weaker response can also be explained by large variety in response induced by the absorption a complex mixture of nutrients.

### Effect of weight loss on fasting metabolism in abdominally obese subjects

The effect of WL on fasting metabolism of abdominally obese subjects only involved two amino acids and creatinine. We have refrained from speculation on the underlying biological mechanism of this minor difference in fasting metabolism. Our observation stands in contrast to the previously observed changes in baseline levels of BCAAs, AAACs, carnitine and metabolites of the TCA cycle (Shah et al. 2012; Wahl et al. 2015). It also contrasts with the observed improvement of insulin sensitivity upon WL in our study population, indicating that the abdominally obese phenotype partially reversed towards a lean one (Newgard et al. 2009; Newgard 2012). Furthermore, we did not observe an effect of WL on C_2_/C_n_ acylcarnitine ratios.

In our study, WL affected the expression of 835 genes in the fasting state. WL resulted in lower expression of genes involved in oxidative phosphorylation PBMCs at baseline. These genes also show lower expression levels in lean compared to abdominally obese individuals (Fig. 3). Furthermore, baseline expression of genes involved in carbohydrate metabolism is different between lean and abdominally obese subject. However, the expression levels of these genes do not seem to change towards the expression levels observed in the lean upon WL. Moreover, gene sets involved in immune regulation were higher expressed after WL, while these gene sets were enriched in lean subjects compared to abdominally obese subjects before the intervention. A possible explanation for the observed differences in expressions of immune-related genes is a shift in cell population occurring during the study. Unfortunately, differentiated blood cell counts were not performed in the current study so we cannot confirm this. Therefore, we cannot conclude that the observed differences are due to a difference in immune status between lean and abdominally obese.

We also observed significant differences in the control group, which was not expected since our control group matched in BMI and waist circumference at the start of the intervention and the control group did not lose any weight. These differences suggest a seasonal effect; however, due to the design of the study this explanation is unlikely. Our remaining explanation is that the participants in the control group changed their behaviour during the course of the study although they were asked to maintain habitual alcohol consumption and physical activity and did report any change in these parameters (Joris et al. 2016). Such change in behaviour of participants in the control group is not uncommon, as described in a systematic review of WL intervention trials (Waters et al. 2012).

### Effect of weight loss on postprandial metabolic response in abdominally obese subjects

The predominant effect of the WL intervention on the postprandial response in abdominally obese subjects involved three amino acids, five oxylipins, choline, creatine and glucose. Several differences in the postprandial response that were observed between lean and abdominally obese subjects, however, were not observed after WL in the abdominally obese. Lower glucose levels after WL suggests that the mixed meal challenge stimulates a more prolonged insulin response, which is in line with previous observations (Stroeve et al. 2015). In our study the WL intervention induced a small number of shifts in fasting metabolite level; the impact of the mixed meal on the postprandial metabolic response involved more metabolites but was still subtle. This is in line with Geidenstam et al. who also found only a modest number of differences in the postprandial metabolic response upon OGTT, mostly branched and aromatic acids, after WL and a maintenance period in abdominally obese subjects. The authors attribute this to the heterogeneity in the postprandial response of abdominally obese to an OGTT challenge. The heterogeneity in postprandial response to a mixed meal most likely also explains why also in our study only modest postprandial effects of WL were observed. In contrast, a recent study by Kardinaal et al. (2015), suggested that a change in challenge response is a more sensitive biomarker of metabolic resilience than changes in fasting metabolism. This discrepancy might be due to either their particular study population, which consisted of a homogeneous group of males with metabolic syndrome, whereas within our study the subjects were a more heterogeneous group of healthy abdominally obese. The study from Kardinaal et al. also used a high fat challenge, which may provoke a more targeted postprandial response as the mixed meal used in our study. In our study the strong and rapid direct uptake of amino acids and fatty acids from the mixed meal may have shrouded observation of postprandial metabolic differences. Therefore the recent recommendation of a mixed meal as the optimal challenge for demonstrating subtle improvements in metabolic flexibility needs to be expounded with regard to the composition of such a challenge (Stroeve et al. 2015).

Genes related to carbohydrate metabolism were downregulated in abdominally obese subjects upon a mixed meal challenge after WL compared to before the WL intervention. Figure 3 shows that the reduced response of genes in the carbohydrate metabolism to the challenge results in expression levels that are more comparable to expression levels observed in lean subjects. As insulin sensitivity is also improved after WL the attenuation of the expression of genes in carbohydrate metabolism seems consistent with this improvement in insulin sensitivity. Similarly, genes related to inflammatory pathways also showed a dampened response in the abdominally obese after WL, which is also more in line with the response observed in lean subjects. Overall, we conclude that the postprandial responses to WL seem to shift towards the lean response after WL with regard to carbohydrate metabolism and the inflammatory response. Although the observed shift suggests that subjects after WL have a leaner phenotype, they do not reach the level of lean subjects, which is in line with the actual WL. Furthermore, at the end of the study the subjects were maintaining weight. This may result in a less pronounced response when comparing to studies in which subjects were still losing weight at the time of measurement.

## Conclusion

Our results are in line with recent observations that the metabolic phenotype of abdominally obese and lean subjects is different with respect to both the plasma metabolome and PBMC gene expression in the fasting state. The difference in phenotypic flexibility between lean and abdominally obese was also reflected in the mixed meal postprandial response of the plasma metabolome and PBMC gene expression. The difference in response of a downstream metabolite of a branched chain amino acid suggests compromised function of the BCKDH complex in the abdominally obese. The metabolites and genes that differ at baseline show minor overlap with those that are different in their postprandial response. This indicates that the mechanisms accounting for the observed differences between lean and abdominally obese in the fasted state are different from the mechanisms underlying the differences during the postprandial mixed meal response.

Compared to the difference in lean-abdominally obese metabolic phenotype, WL had a small effect on the fasting plasma metabolome. Also, the effect of WL on baseline gene expression in PBMCs was smaller; the main effect was a shift of genes related to oxidative phosphorylation towards the lean phenotype. The impact of WL on the mixed meal postprandial response of plasma metabolites and PBMC gene expression was modest and mainly point to altered enzymatic lipid oxidation and carbohydrate metabolism upon WL. The WL induced shifts of baseline and postprandial response of plasma metabolome and PBMC gene expression showed little overlap with the differences between lean-abdominally obese metabolic phenotype.

The modest number of significant differences in postprandial metabolic responses between lean-abdominally obese and abdominally obese before and after WL may be explained by the complex composition of the mixed meal. Hence, the composition of meal challenges should be considered carefully in order to provoke a distinct postprandial metabolic response.

## Electronic supplementary material

Below is the link to the electronic supplementary material.

Supplementary material 1 (DOCX 869 KB)
