# Supplementary material for: Weight loss moderately affects the mixed meal challenge response of the plasma metabolome and transcriptome of peripheral blood mononuclear cells in abdominally obese subjects
Source: Metabolomics. 2018 Mar 5;14(4):46. doi: 10.1007/s11306-018-1328-x (PMC5838115; doi:10.1007/s11306-018-1328-x)
Supplement: Supplementary file 1 — Supplementary material 1 (DOCX 869 KB) [file 11306_2018_1328_MOESM1_ESM.docx]

**Supplementary Material**

**Postprandial metabolic response in lean and abdominally obese subjects**

Despite the significant inter-individual variation between the time-responses, differentiating features can be recognized according to four dominant postprandial metabolic events [1, 2] : glycolysis, lipolysis, ketogenesis, proteolysis and amino acid oxidation.

*Glycolysis* In lean volunteers the mixed meal provoked a prolonged insulin response, in line with previous observations [3]. As a consequence postprandial effects on glycolysis, lipolysis and ketogenesis can be expected [1]. Indeed, the initial postprandial increase of glucose was rapidly followed by a strong insulin-induced decrease. This pattern was also observed for lactate [1] and downstream TCA metabolites [2]. The postprandial response of glucose did not differ significantly between the lean and abdominally obese group (lFDR> 0.2), which was also the case for most downstream TCA metabolites, except for citrate.

*Lipolysis* The essential fatty acids linoleic acid (LA) and alpha linolenic acid (ALA) follow the characteristic insulin-induced non esterified fatty acid response (NEFA), which is a strong decrease followed by an increase after 1 hour (Supplementary Figure S3-a ). Hence in our study mixed meal challenge did not induce the lag phase response for longer chain (C_>16_) fatty acids [2] that was previously observed for another mixed meal challenge. Apparently, for mixed meal challenge in our study, insulin-induced depletion of FFAs is dominant over intake of FFAs. Also the FFA response did not differ significantly between the lean and abdominally obese group.

Within this study we assessed oxylipins since they have been associated with a range of inflammatory mechanisms. The enzymatic oxidation pathways of the oxylipins are known, but our attempt to model their postprandial response with a recently introduced first order kinetic model [4] was not successful. Only modest correlations between oxylipins and their precursors were found (Supplementary Table S7) which we attribute this to complexity of having both an insulin effect combined with dietary intake of fatty acids in the mixed meal challenge. The postprandial response of several oxylipins was however different between the lean an abdominally obese group, which suggests differences in lipid oxidation between the abdominally obese and lean groups.

Once the free fatty acids (FFAs) are destined as fuel they enter the mitochondria as acylcarnitines in order to undergo stepwise β-oxidation to acetylcarnitine which enters the TCA cycle for energy production. Plasma levels of fatty acid derived acylcarnitines (FAAC, Supplementary Table S8) reflect increased FFA availability and/or a shift in β-oxidation flux. In the lean subjects, the mixed meal challenge induced a monotonous postprandial decrease of both fatty acid derived acylcarnitines and acetylcarnitine, in line with reduced availability of FFAs and/or reduced β-oxidation upon switching from b-oxidation to glycolysis [5]. The ratio between C_2_ (acetyl) carnitine and fatty acid derived acylcarnitines has been proposed as direct readouts for intracellular β-oxidation [6]. During the postprandial phase we observe that for the C_2_/FAAC ratios interindividual difference is more pronounced at fasting rather than response (Supplementary Figure S3-b). We could however not observe a difference between the abdominally obese and lean groups for these C_2_/FAAC rations.

Nadir acylcarnitine levels have been proposed as a single parameter summary of their postprandial response [7]. We found correlations with phenotypical parameters such as HOMA and lean body mass (Supplementary Table S9). Although this suggests that postprandial acylcarnitine responses are related to fatty acid oxidation flux, we could not observe statistically significant differences between the lean and abdominally obese group.

*Ketogenesis* In lean volunteers we observe a monotonous postprandial decrease of the ketone bodies β-hydroxybutyric acid, and acetone (Supplementary Figure S3-c ) which is in line with previous observations upon administration of mixed meal challenge[2]. The decrease can be explained by direct inhibition of ketogenesis by insulin and indirectly by decrease of FFA due to inhibition of lipolysis. Acetoacetate however, goes up due to enhanced postprandial amino acid flux (Figure 1). In the abdominally obese none of the ketone bodies showed a postprandial response.

*Proteolysis and amino acid oxidation* Upon administration of the mixed meal challenge insulin-induced inhibition of proteolysis is expected. An indication for an impact on muscle metabolism is the weak postprandial decrease in creatinine for lean subjects (Supplementary S5-d), which is in contrast to a previous observation [2]. The postprandial response in the abdominally obese did however not differ significantly from[8] the lean group.

Unfortunately, observation at the level of amino acids is obscured by direct dietary uptake upon the mixed meal challenge [8]. Nevertheless, still several amino acids differ in their postprandial response in lean vs abdominally obese, in line with a recent study [8]. In this study these effects were attributed to impairment of the TCA cycle in abdominally obese, this was confirmed by differences in postprandial response of citrate and succinate.

The mixed meal challenge induces a strong postprandial increase of BCAA which can be explained by direct uptake and transamination of other dietary amino acids in the splanchnic bed [9]. After the steep increase of BCAAs, their levels drop rapidly due to insulin-induced uptake in muscle or other tissues[10]. Within the muscle, BCAAs (leucine in particular) are rapidly transaminated to branched chain α-keto acids (BCKAs) and excess nitrogen is subsequently released as glutamine. Indeed we observed a second peak in the glutamine response curve which we attribute to BCAA’s transferring their amine group (Supplementary Figure S3-e ) [11]. The BCKAs are oxidized to C_4-5_-CoA derivatives of CoA to finally produce acetoacetate, C_2_(acetyl)-CoA and C_3_ (proprionyl)-CoA, all of which can enter the TCA cycle [10]. Excess levels of the C_3-5_ fatty acids appear in plasma as amino acid derived acylcarnitines (AAAC, Supplementary Table S3) which can be considered as proxies of BCAA oxidation. The kinetic profile of the AAACs generally follows the BCAA profile (only propionyl and isovaleryl with a bit delay compare to BCAAs. During the postprandial phase we observe that for the BCAA/AAAC ratios inter-individual differences are more pronounced at fasting rather than for their postprandial response (Supplementary Figure S3-f). The postprandial response of the BCAAs and AAACs does not differ between lean and abdominally obese, but interestingly we observe significant differences in the response of two branched chain keto acids (α-ketoisovaleric acid, 2-hydroxyisovalerate) and methylmalonic acid (Supplementary Figure S3-g). This indicates that in the abdominally obese group oxidation of mitochondrial BCAA by the BCKDH complex might be impaired, causing temporary postprandial accumulation of these downstream BCAAs catabolites[12].

**Figure S1.** Flow diagram showing the number of genes of which the expression was significantly different between lean(n=15) and abdominally obese (n=29) subjects at fasting, the number of genes that changed in expression after a mixed meal challenge and the number of genes that changed differently in expression in abdominally obese relative to the lean subjects after a mixed meal challenge. A change was significant if *P*< 0.05.

**Figure S2.** Flow diagram showing the number of genes of which the expression was significantly different in abdominally obese (n=29) before weight loss (D1) in response to mixed meal challenge, the number of genes that changed significantly in expression before (D1) and after (D2) intervention (WL, CRTL) in response to mixed meal challenge, the number of genes that changed significantly at T0 comparing subjects in WL or CTRL before and after intervention. A change was significant if *P*< 0.05.


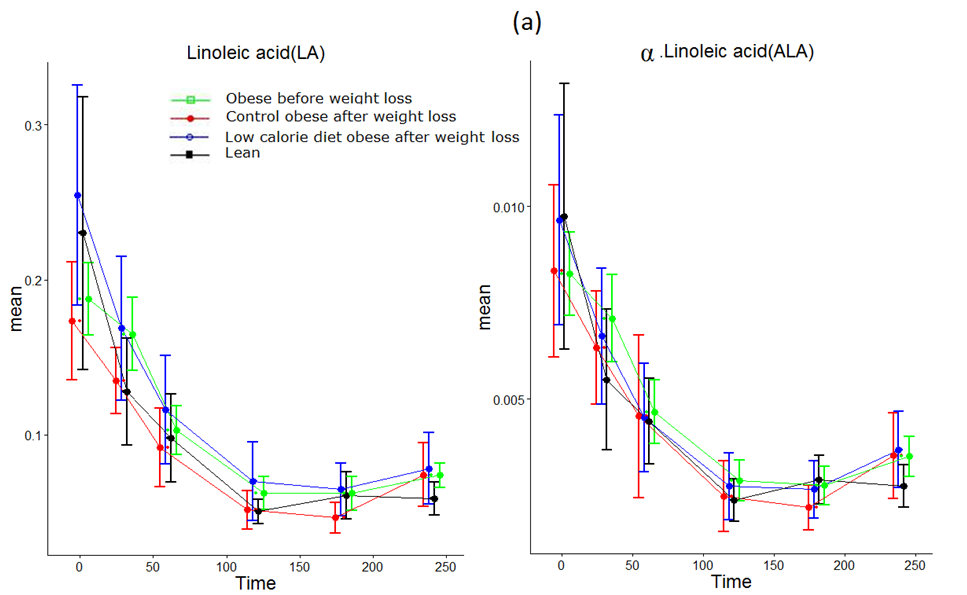


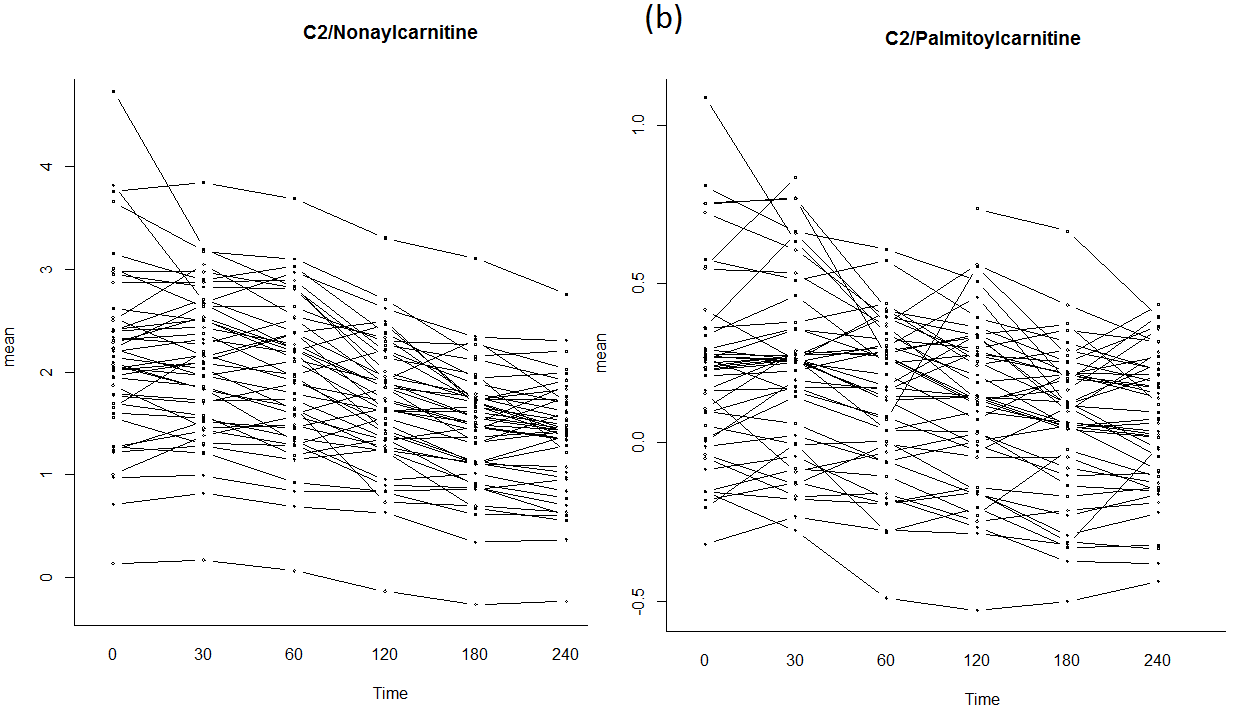


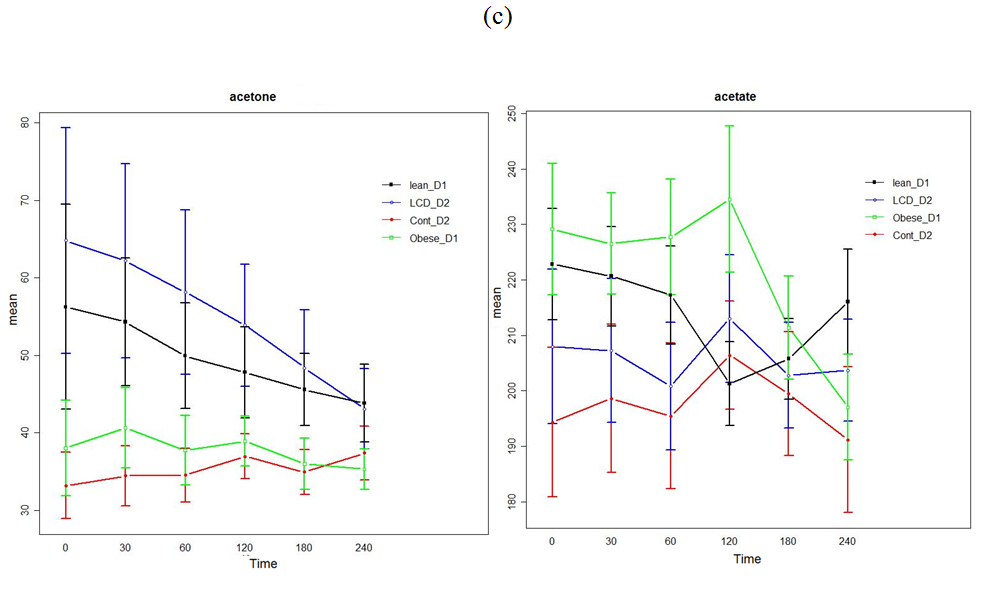


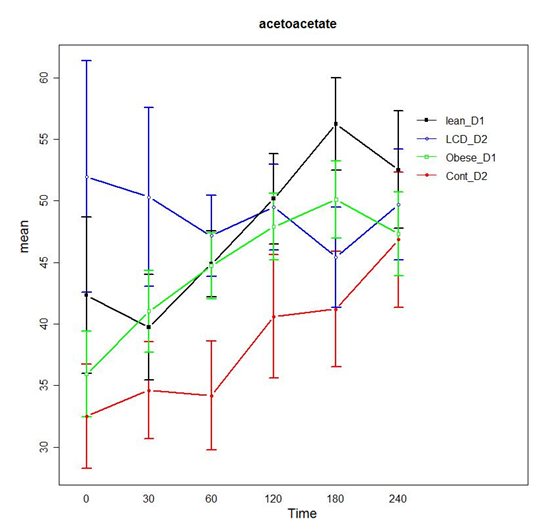


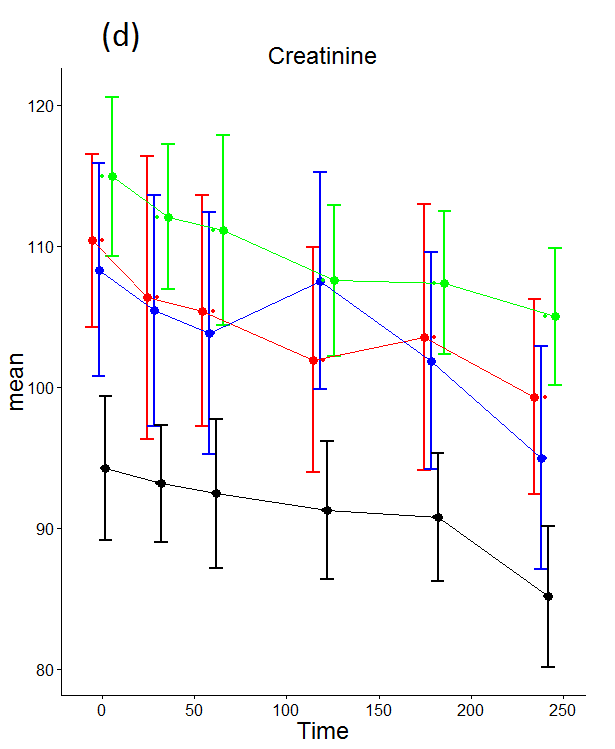


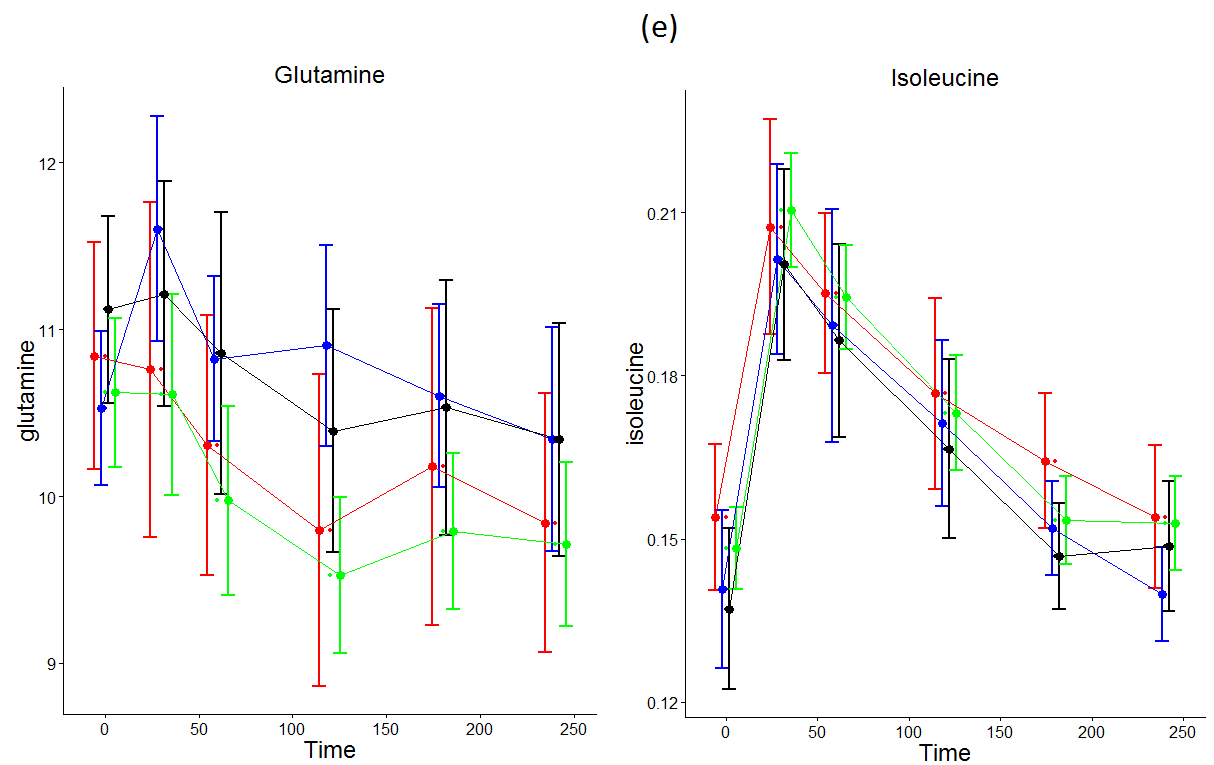


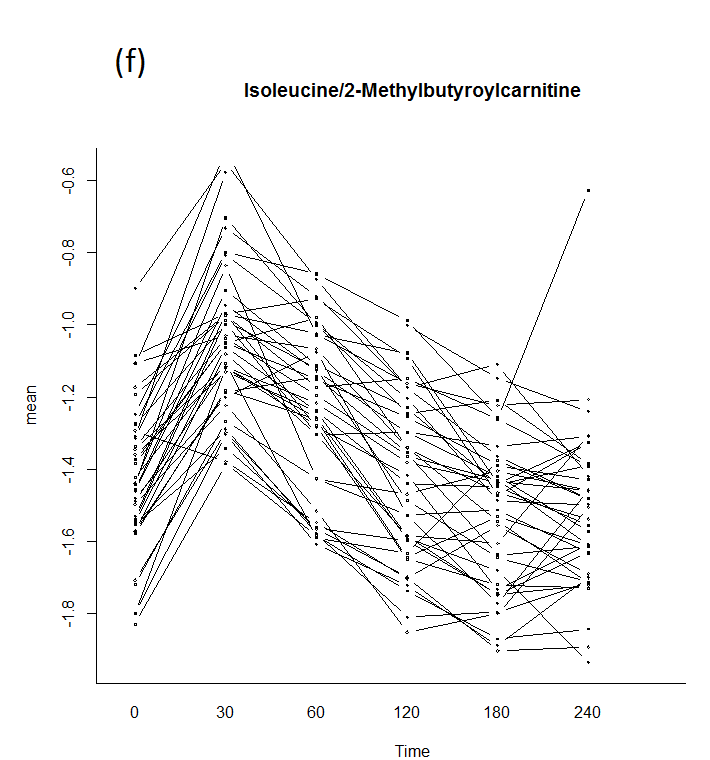


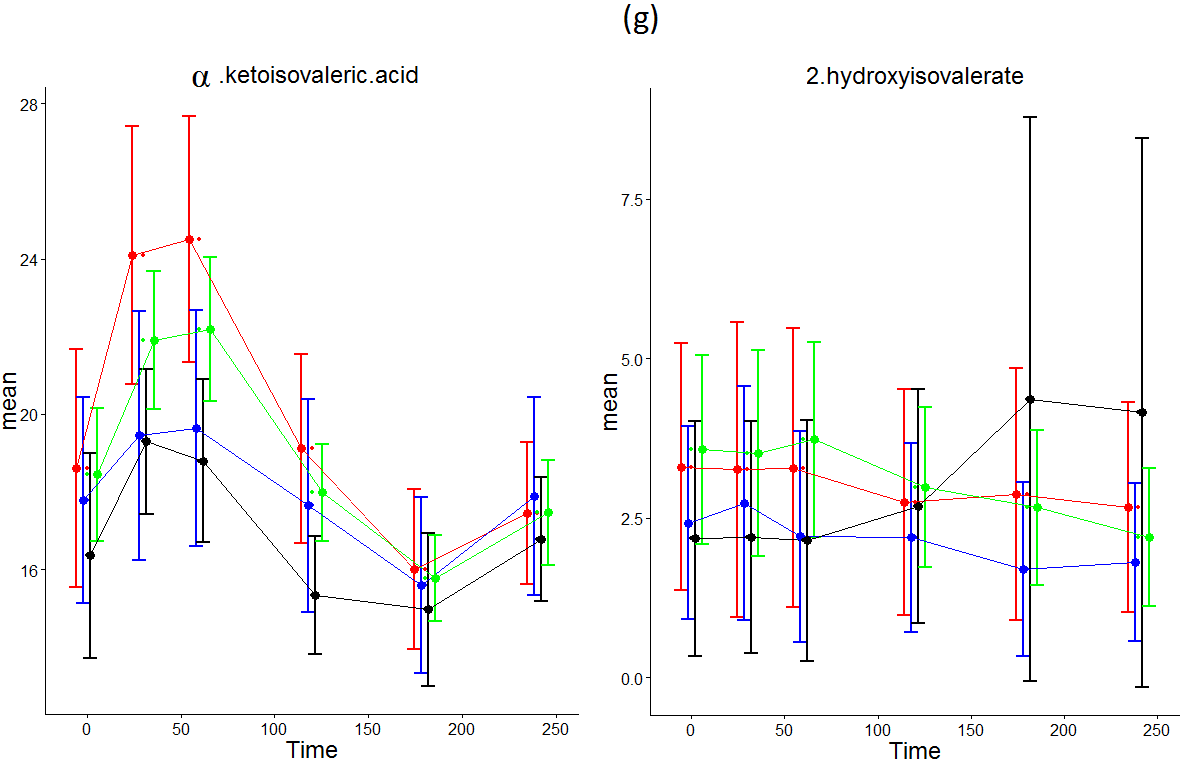


**Figure S3.** Mean postprandial response curves for linoleic acid and α-linolenic acid (a). Mean postprandial curve (lean subjects only) of ratio between acetylcarnitine (c2) and fatty acid derived acylcarnitines; each color indicates one individual (b). Mean postprandial curve of ketone bodies (c), carnitine (d), glutamine and isoleucine (e). Individual plot (lean subjects only) of ratio between Isoleucine and 2-ethylbutyroylcarnitine; each color indicate one individual (f); Mean postprandial curve for alpha ketoisovaleric acid and 2-hydroxyisovalerate (g).

**Table S1.** Overview of significantly (FDR<0.2) positively /negatively enriched gene sets between abdominally obese vs. lean subjects at fasting (T0).

**Positively enriched gene sets**

| NAME | SIZE | NES | FDR q-val |
| --- | --- | --- | --- |
| REACT_RESPIRATORY ELECTRON TRANSPORT | 56 | 2.08 | 0.01 |
| WIP_HS_OXIDATIVE_PHOSPHORYLATION | 46 | 2.03 | 0.02 |
| KEGG_OXIDATIVE PHOSPHORYLATION | 92 | 2.02 | 0.01 |
| REACT_RESPIRATORY ELECTRON TRANSPORT | 69 | 2.00 | 0.01 |
| WIP_HS_ELECTRON_TRANSPORT_CHAIN | 76 | 1.92 | 0.04 |
| KEGG_PARKINSON'S DISEASE | 86 | 1.91 | 0.04 |
| REACT_DESTABILIZATION OF MRNA BY KSRP | 15 | 1.84 | 0.08 |
| REACT_DEADENYLATION-DEPENDENT MRNA DECAY | 39 | 1.84 | 0.07 |
| KEGG_RNA DEGRADATION | 60 | 1.80 | 0.11 |

**Negatively enriched gene sets**

| NAME | SIZE | NES | FDR q-val |
| --- | --- | --- | --- |
| NCI_IL23PATHWAY | 18 | -2.08 | 0.08 |
| WIP_HS_NOTCH_SIGNALING_PATHWAY | 24 | -2.00 | 0.09 |
| BIOC_IL1RPATHWAY | 22 | -1.89 | 0.20 |
| NCI_ATF2_PATHWAY | 27 | -1.86 | 0.18 |
| BIOC_KERATINOCYTEPATHWAY | 35 | -1.84 | 0.18 |
| BIOC_NTHIPATHWAY | 20 | -1.81 | 0.20 |
| WIP_HS_OXIDATIVE_STRESS | 18 | -1.79 | 0.19 |
| KEGG_OSTEOCLAST DIFFERENTIATION | 94 | -1.77 | 0.20 |
| NCI_AP1_PATHWAY | 36 | -1.77 | 0.18 |

**Table S2.** Overview of significantly (FDR<0.2) up- and down-regulated gene sets during postprandial mixed meal challenge response between abdominally obese and lean subjects.

**Upregulated gene sets**

| NAME | SIZE | NES | FDR  q-val | Direction |
| --- | --- | --- | --- | --- |
|  |  |  |  | obese Lean |
| NCI_IL6_7PATHWAY | 35 | 2.13 | 0.01 | up down |
| NCI_IFNGPATHWAY | 35 | 2.01 | 0.04 | up down |
| KEGG_LYSOSOME | 97 | 2.01 | 0.03 | up down |
| KEGG_TUBERCULOSIS | 114 | 2.00 | 0.02 | up down |
| WIP_HS_TYPE_II_INTERFERON_SIGNALING_(IFNG) | 27 | 1.99 | 0.02 | up down |
| KEGG_STAPHYLOCOCCUS AUREUS INFECTION | 28 | 1.99 | 0.02 | up down |
| KEGG_PORPHYRIN AND CHLOROPHYLL METABOLISM | 18 | 1.98 | 0.02 | up down |
| KEGG_INFLUENZA A | 121 | 1.97 | 0.02 | up down |
| KEGG_LEISHMANIASIS | 51 | 1.96 | 0.02 | up down |
| KEGG_PERTUSSIS | 43 | 1.94 | 0.02 | up down |
| KEGG_STARCH AND SUCROSE METABOLISM | 20 | 1.89 | 0.04 | up down |
| WIP_HS_EPITHELIUM_TARBASE | 200 | 1.89 | 0.04 | up down |
| NCI_ENDOTHELINPATHWAY | 36 | 1.89 | 0.03 | up down |
| REACT_TOLL LIKE RECEPTOR 7_8 (TLR7_8) CASCADE | 60 | 1.89 | 0.03 | up down |
| REACT_MYD88 DEPENDENT CASCADE INITIATED ON ENDOSOME | 60 | 1.89 | 0.03 | up down |
| NCI_UPA_UPAR_PATHWAY | 19 | 1.89 | 0.03 | up down |
| REACT_TOLL LIKE RECEPTOR 9 (TLR9) CASCADE | 62 | 1.87 | 0.03 | up down |
| KEGG_SALMONELLA INFECTION | 52 | 1.86 | 0.04 | up down |
| WIP_HS_PROSTAGLANDIN_SYNTHESIS_AND_REGULATION | 16 | 1.86 | 0.04 | up down |
| REACT_METABOLISM OF CARBOHYDRATES | 88 | 1.84 | 0.04 | up down |
| KEGG_PHAGOSOME | 94 | 1.83 | 0.05 | up down |
| REACT_TRAF6 MEDIATED INDUCTION OF  NFKB AND MAP KINASES UPON TLR7_8 OR 9 ACTIVATION | 59 | 1.82 | 0.05 | up down |
| NCI_ANTHRAXPATHWAY | 16 | 1.82 | 0.05 | up down |
| NCI_ATF2_PATHWAY | 27 | 1.81 | 0.05 | up down |
| REACT_GOLGI ASSOCIATED VESICLE BIOGENESIS | 42 | 1.81 | 0.05 | up down |
| REACT_CLATHRIN DERIVED VESICLE BUDDING | 49 | 1.81 | 0.05 | up down |
| REACT_TRANS-GOLGI NETWORK VESICLE BUDDING | 49 | 1.80 | 0.05 | up down |
| KEGG_OSTEOCLAST DIFFERENTIATION | 94 | 1.78 | 0.06 | up down |
| BIOC_KERATINOCYTEPATHWAY | 35 | 1.78 | 0.06 | down down |
| WIP_HS_IL-6_SIGNALING_PATHWAY | 35 | 1.78 | 0.06 | up down |
| KEGG_NEUROACTIVE LIGAND-RECEPTOR INTERACTION | 38 | 1.77 | 0.06 | up down |
| WIP_HS_LEUKOCYTE_TARBASE | 107 | 1.76 | 0.07 | up down |
| **Table S2. Upregulated gene sets continued** |  |  |  |  |
| REACT_MEMBRANE TRAFFICKING | 97 | 1.75 | 0.07 | up down |
| BIOC_TIDPATHWAY | 15 | 1.75 | 0.07 | up down |
| NCI_TOLL_ENDOGENOUS_PATHWAY | 19 | 1.75 | 0.07 | up down |
| WIP_HS_OXIDATIVE_STRESS | 18 | 1.74 | 0.08 | up down |
| KEGG_COMPLEMENT AND COAGULATION CASCADES | 15 | 1.74 | 0.08 | up down |
| NCI_HES_HEYPATHWAY | 28 | 1.74 | 0.07 | up down |
| BIOC_IL1RPATHWAY | 22 | 1.73 | 0.08 | up down |
| REACT_P75 NTR RECEPTOR-MEDIATED SIGNALLING | 62 | 1.73 | 0.07 | up down |
| REACT_MAP KINASE ACTIVATION IN TLR CASCADE | 42 | 1.73 | 0.08 | up down |
| WIP_HS_REGULATION_OF_TOLL-LIKE_RECEPTOR_SIGNALING_PATHWAY | 96 | 1.72 | 0.08 | up down |
| KEGG_MALARIA | 23 | 1.72 | 0.07 | up down |
| NCI_LYSOPHOSPHOLIPID_PATHWAY | 38 | 1.72 | 0.07 | up down |
| KEGG_FRUCTOSE AND MANNOSE METABOLISM | 22 | 1.71 | 0.08 | up down |
| WIP_HS_SENESCENCE_AND_AUTOPHAGY | 61 | 1.69 | 0.10 | up down |
| WIP_HS_SQUAMOUS_CELL_TARBASE | 94 | 1.69 | 0.10 | up down |
| REACT_CYTOKINE SIGNALING IN IMMUNE SYSTEM | 154 | 1.69 | 0.10 | up down |
| REACT_GLUCOSE METABOLISM | 44 | 1.68 | 0.10 | up down |
| NCI_HNF3APATHWAY | 18 | 1.68 | 0.10 | up down |
| REACT_ASSOCIATION OF TRIC_CCT WITH TARGET  PROTEINS DURING BIOSYNTHESIS | 23 | 1.67 | 0.11 | up down |
| KEGG_FATTY ACID METABOLISM | 26 | 1.67 | 0.10 | down down |
| WIP_HS_NOD_PATHWAY | 28 | 1.67 | 0.10 | up down |
| REACT_INTERLEUKIN-1 SIGNALING | 34 | 1.67 | 0.11 | up down |
| REACT_ERK_MAPK TARGETS | 17 | 1.66 | 0.11 | up down |
| REACT_MAPK TARGETS_ NUCLEAR EVENTS MEDIATED  BY MAP KINASES | 25 | 1.66 | 0.11 | up down |
| WIP_HS_PHYSIOLOGICAL_AND_PATHOLOGICAL_  HYPERTROPHY_OF_THE_HEART | 17 | 1.65 | 0.11 | down down |
| KEGG_PENTOSE PHOSPHATE PATHWAY | 19 | 1.65 | 0.12 | up down |
| REACT_TRAF6 MEDIATED INDUCTION OF PROINFLAMMATORY CYTOKINES | 51 | 1.65 | 0.12 | up down |
| KEGG_GALACTOSE METABOLISM | 17 | 1.64 | 0.12 | up down |
| WIP_HS_IL-3_SIGNALING_PATHWAY | 37 | 1.63 | 0.12 | up down |
| REACT_SIGNALING BY INTERLEUKINS | 85 | 1.63 | 0.13 | up down |
| WIP_HS_GPCR_LIGAND_BINDING | 24 | 1.63 | 0.13 | up down |
| REACT_INTERFERON SIGNALING | 75 | 1.63 | 0.12 | up down |
| REACT_NRAGE SIGNALS DEATH THROUGH JNK | 30 | 1.62 | 0.13 | up down |
| REACT_HEXOSE TRANSPORT | 33 | 1.62 | 0.13 | up down |
| NCI_P38ALPHABETADOWNSTREAMPATHWAY | 29 | 1.62 | 0.13 | up down |
| KEGG_SPHINGOLIPID METABOLISM | 23 | 1.62 | 0.13 | up down |
| NCI_GMCSF_PATHWAY | 31 | 1.62 | 0.13 | up down |
| WIP_HS_KIT_RECEPTOR_SIGNALING_PATHWAY | 39 | 1.62 | 0.13 | up down |
|  |  |  |  |  |
| **Table S2. Upregulated gene sets continued** |  |  |  |  |
| KEGG_TOLL-LIKE RECEPTOR SIGNALING PATHWAY | 69 | 1.62 | 0.13 | up down |
| NCI_MAPKTRKPATHWAY | 27 | 1.61 | 0.13 | up down |
| REACT_GLUCOSE TRANSPORT | 33 | 1.61 | 0.13 | up down |
| KEGG_FC GAMMA R-MEDIATED PHAGOCYTOSIS | 73 | 1.61 | 0.13 | up down |
| NCI_RB_1PATHWAY | 42 | 1.61 | 0.13 | up down |
| KEGG_RHEUMATOID ARTHRITIS | 48 | 1.60 | 0.14 | up down |
| BIOC_NTHIPATHWAY | 20 | 1.59 | 0.14 | up down |
| WIP_HS_IL-5_SIGNALING_PATHWAY | 29 | 1.59 | 0.14 | up down |
| WIP_HS_TOLL-LIKE_RECEPTOR_SIGNALING_PATHWAY | 69 | 1.59 | 0.15 | up down |
| REACT_TOLL LIKE RECEPTOR 3 (TLR3) CASCADE | 56 | 1.59 | 0.15 | up down |
| REACT_INFLAMMASOMES | 16 | 1.58 | 0.15 | up down |
| KEGG_PPAR SIGNALING PATHWAY | 22 | 1.58 | 0.15 | up down |
| NCI_ARF_3PATHWAY | 19 | 1.58 | 0.15 | up down |
| REACT_INTERFERON GAMMA SIGNALING | 54 | 1.57 | 0.15 | up down |
| NCI_RAC1_PATHWAY | 44 | 1.57 | 0.16 | up down |
| REACT_INTERFERON ALPHA_BETA SIGNALING | 43 | 1.57 | 0.16 | up down |
| KEGG_ADIPOCYTOKINE SIGNALING PATHWAY | 40 | 1.57 | 0.16 | up down |
| KEGG_NEUROTROPHIN SIGNALING PATHWAY | 88 | 1.56 | 0.17 | up down |
| WIP_HS_MRNA_PROCESSING | 114 | 1.55 | 0.17 | up down |
| WIP_HS_FOLATE_METABOLISM | 29 | 1.55 | 0.17 | up down |
| WIP_HS_LYMPHOCYTE_TARBASE | 305 | 1.55 | 0.17 | up down |
| REACT_RECYCLING PATHWAY OF L1 | 26 | 1.55 | 0.17 | up down |
| BIOC_HIVNEFPATHWAY | 45 | 1.55 | 0.17 | up down |
| KEGG_NOD-LIKE RECEPTOR SIGNALING PATHWAY | 43 | 1.55 | 0.17 | up down |
| REACT_NUCLEAR EVENTS (KINASE AND TRANSCRIPTION  FACTOR ACTIVATION) | 20 | 1.54 | 0.17 | up down |
| WIP_HS_APOPTOTIC_EXECUTION_PHASE | 22 | 1.54 | 0.18 | up down |
| KEGG_GLYCOLYSIS _ GLUCONEOGENESIS | 33 | 1.54 | 0.18 | up down |
| WIP_HS_INTRINSIC_PATHWAY_FOR_APOPTOSIS | 16 | 1.54 | 0.17 | up down |
| NCI_IL8CXCR1_PATHWAY | 22 | 1.54 | 0.18 | up down |
| REACT_G ALPHA (12_13) SIGNALLING EVENTS | 47 | 1.54 | 0.18 | up down |
| NCI_CDC42_PATHWAY | 52 | 1.54 | 0.18 | up down |
| REACT_CHAPERONIN-MEDIATED PROTEIN FOLDING | 33 | 1.53 | 0.19 | up down |
| WIP_HS_TNF-ALPHA-NF-KB_SIGNALING_PATHWAY | 161 | 1.52 | 0.19 | up down |
| NCI_TXA2PATHWAY | 40 | 1.52 | 0.19 | up down |
| NCI_CERAMIDE_PATHWAY | 37 | 1.52 | 0.19 | up down |
| REACT_L1CAM INTERACTIONS | 50 | 1.52 | 0.19 | up down |
| KEGG_AMOEBIASIS | 40 | 1.52 | 0.19 | up down |
| NCI_HIF1_TFPATHWAY | 44 | 1.52 | 0.19 | up down |
| KEGG_BLADDER CANCER | 21 | 1.51 | 0.19 | up down |
| NCI_RHOA_REG_PATHWAY | 25 | 1.51 | 0.19 | up down |
| NCI_KITPATHWAY | 37 | 1.51 | 0.19 | up down |
| **Table S2. Upregulated gene sets continued** |  |  |  |  |
| NCI_RAC1_REG_PATHWAY | 28 | 1.51 | 0.19 | up down |
| NCI_S1P_S1P3_PATHWAY | 20 | 1.51 | 0.19 | up down |

**Downregulated gene sets**

| NAME | SIZE | NES | FDR q-val | Direction |
| --- | --- | --- | --- | --- |
|  |  |  |  | obese Lean |
| NCI_CD8TCRPATHWAY | 47 | -2.12 | 0.02 | down up |
| REACT_GENERATION OF SECOND MESSENGER  MOLECULES | 29 | -1.91 | 0.13 | down up |
| REACT_BRANCHED-CHAIN AMINO ACID CATABOLISM | 15 | -1.87 | 0.12 | down up |
| REACT_BIOSYNTHESIS OF THE N-GLYCAN PRECURSOR | 24 | -1.83 | 0.14 | down up |
| WIP_HS_GENERIC_TRANSCRIPTION_PATHWAY | 15 | -1.81 | 0.12 | down up |
| NCI_TCR_PATHWAY | 58 | -1.70 | 0.26 | down up |
| KEGG_T CELL RECEPTOR SIGNALING PATHWAY | 81 | -1.69 | 0.24 | down up |
| NCI_CD8TCRDOWNSTREAMPATHWAY | 46 | -1.69 | 0.22 | down up |
| WIP_HS_INFLAMMATORY_RESPONSE_PATHWAY | 15 | -1.68 | 0.20 | down up |
| BIOC_AMIPATHWAY | 17 | -1.67 | 0.20 | up up |
| KEGG_ABC TRANSPORTERS | 19 | -1.67 | 0.18 | up up |
| REACT_TCR SIGNALING | 57 | -1.66 | 0.18 | up up |
| REACT_GENERIC TRANSCRIPTION PATHWAY | 134 | -1.65 | 0.18 | down up |
| BIOC_CSKPATHWAY | 17 | -1.62 | 0.19 | down up |
| NCI_IL2_STAT5PATHWAY | 25 | -1.61 | 0.20 | up up |

Size indicates the number of total genes which are involve in the corresponding pathway. (NES):

normalised enrichment scores and (FDR): false discovery rate.

**Table S3.** Correlation of the fasting plasma metabolome at baseline with HOMA

| **A** | **Acylcarnitine** | **C_2_/C_n vs._ HOMA** | | **C_2vs._ HOMA** | | **C_n vs._ HOMA** | |
| --- | --- | --- | --- | --- | --- | --- | --- |
|  | C_n_ | *P* | ρ | *P* | ρ | *P* | ρ |
|  | AAAC |  |  |  |  |  |  |
|  | C_4_ (butyryl) | <0.001 | -0.44 | 0.70 | -0.04 | <0.001 | 0.41 |
|  | C_3_ (propionyl) | 0.01 | -0.30 |  |  | 0.003 | 0.38 |
|  | C_5_(2-methylbutyroyl) | 0.09 | -0.21 |  |  | 0.02 | 0.28 |
|  | C_0_ (carnitine) | 0.1 | -0.20 |  |  | 0.07 | 0.22 |
|  | FAAC |  |  |  |  |  |  |
|  | C_6_ (hexanoyl) | <0.001 | -0.55 |  |  | 0.01 | 0.31 |
|  | C_18_ (stearoyl) | 0.4 | -0.10 |  |  | 0.9 | 0.01 |
|  | C_16_ (palmitoyl) | 0.07 | -0.22 |  |  | 0.1 | 0.20 |
|  | C_14_ (myristol) | 0.1 | -0.20 |  |  | 0.3 | 0.10 |
| **B** |  |  |  |  |  | ***AA vs. HOMA*** | |
|  | **BCAA** |  |  |  |  | *P* | ρ |
|  | Isoleucine |  |  |  |  | 0.06 | 0.34 |
|  | Leucine |  |  |  |  | 0.02 | 0.30 |
|  | Valine |  |  |  |  | 0.02 | 0.30 |
|  | **Amino acids** |  |  |  |  | *P* | ρ |
|  | Tyrosine |  |  |  |  | <0.001 | 0.57 |
|  | Phenyl alanine |  |  |  |  | 0.02 | 0.30 |
|  | 2aminoadipic acid |  |  |  |  | 0.06 | 0.24 |

AAAC: amino acid derived acylcarnitine, FAAC: fatty acid derived acylcarnitines. AA: amino acid.

ρ: Spearman's rank correlation coefficient. Fasting levels (T0) of all subjects (lean and abdominally obese) before

and after interventions were included.

**Table S4.** Overview of significantly (FDR<0.05) upregulated and downregulated gene sets in WL abdominally obese subjects at fasting (T0) before and after weight loss intervention (D2 vs. D1)

**Upregulated gene sets**

| **NAME** | **SIZE** | **NES** | **FDR q-val** |  |
| --- | --- | --- | --- | --- |
| NCI_IL12_2PATHWAY | 45 | 2.27 | 0 |  |
| KEGG_T CELL RECEPTOR SIGNALING PATHWAY | 81 | 2.14 | 0.01 |  |
| KEGG_T CELL RECEPTOR SIGNALING PATHWAY | 81 | 2.14 | 0.01 |  |
| BIOC_NTHIPATHWAY | 20 | 2.05 | 0.02 |  |
| BIOC_PPARAPATHWAY | 35 | 2.03 | 0.02 |  |
| NCI_FGF_PATHWAY | 26 | 1.99 | 0.03 |  |
| NCI_FCER1PATHWAY | 51 | 1.97 | 0.03 |  |
| REACT_NRAGE SIGNALS DEATH THROUGH JNK | 30 | 1.95 | 0.03 |  |
| REACT_SIGNALING BY RHO GTPASES | 77 | 1.94 | 0.03 |  |
| KEGG_SALMONELLA INFECTION | 52 | 1.94 | 0.03 |  |
| REACT_RHO GTPASE CYCLE | 77 | 1.94 | 0.03 |  |
| BIOC_IL1RPATHWAY | 22 | 1.92 | 0.03 |  |
| NCI_TCRCALCIUMPATHWAY | 18 | 1.91 | 0.03 |  |
| WIP_HS_NOD_PATHWAY | 28 | 1.9 | 0.03 |  |
| KEGG_NEUROTROPHIN SIGNALING PATHWAY | 88 | 1.9 | 0.03 |  |
| NCI_AP1_PATHWAY | 36 | 1.89 | 0.03 |  |
| NCI_IL23PATHWAY | 18 | 1.88 | 0.03 |  |
| REACT_SIGNALING BY GPCR | 152 | 1.88 | 0.03 |  |
| REACT_INTERACTIONS OF THE IMMUNOGLOBULIN SUPERFAMILY (IGSF) | 31 | 1.88 | 0.03 |  |
| KEGG_LEISHMANIASIS | 51 | 1.88 | 0.03 |  |
| KEGG_CARBOHYDRATE DIGESTION AND ABSORPTION | 19 | 1.87 | 0.03 |  |
| NCI_ERBB4_PATHWAY | 20 | 1.86 | 0.03 |  |
| NCI_HIF1_TFPATHWAY | 44 | 1.86 | 0.03 |  |
| KEGG_TOLL-LIKE RECEPTOR SIGNALING PATHWAY | 69 | 1.86 | 0.03 |  |
| KEGG_NOD-LIKE RECEPTOR SIGNALING PATHWAY | 43 | 1.84 | 0.04 |  |
| WIP_HS_RANKL-RANK_SIGNALING_PATHWAY | 41 | 1.84 | 0.04 |  |
| REACT_G ALPHA (12_13) SIGNALLING EVENTS | 47 | 1.84 | 0.04 |  |
| WIP_HS_TOLL-LIKE_RECEPTOR_SIGNALING_PATHWAY | 69 | 1.83 | 0.04 |  |
| NCI_EPHBFWDPATHWAY | 21 | 1.83 | 0.03 |  |
| NCI_ATF2_PATHWAY | 27 | 1.83 | 0.03 |  |
| WIP_HS_INSULIN_SIGNALING | 113 | 1.82 | 0.04 |  |
| NCI_IL1PATHWAY | 26 | 1.82 | 0.04 |  |
| KEGG_OSTEOCLAST DIFFERENTIATION | 94 | 1.81 | 0.04 |  |
| REACT_GPCR DOWNSTREAM SIGNALING | 143 | 1.81 | 0.04 |  |
| NCI_CD8TCRDOWNSTREAMPATHWAY | 46 | 1.8 | 0.04 |  |
| BIOC_KERATINOCYTEPATHWAY | 35 | 1.8 | 0.04 |  |
| NCI_CXCR4_PATHWAY | 78 | 1.8 | 0.04 |  |
| REACT_ION CHANNEL TRANSPORT | 18 | 1.79 | 0.04 |  |
| **Table S4. Upregulated gene sets continued** |  |  |  |  |
| KEGG_MTOR SIGNALING PATHWAY | 36 | 1.79 | 0.04 |  |
| BIOC_TOLLPATHWAY | 28 | 1.79 | 0.04 |  |
| **Downregulated gene sets**   \| **NAME** \| **SIZE** \| **NES** \| **FDR q-val** \| \| --- \| --- \| --- \| --- \| \| KEGG_PARKINSON'S DISEASE \| 86 \| -2.5 \| 0 \| \| REACT_RESPIRATORY ELECTRON TRANSPORT \| 56 \| -2.45 \| 0 \| \| KEGG_OXIDATIVE PHOSPHORYLATION \| 92 \| -2.42 \| 0 \| \| WIP_HS_OXIDATIVE_PHOSPHORYLATION \| 46 \| -2.36 \| 0 \| \| WIP_HS_ELECTRON_TRANSPORT_CHAIN \| 76 \| -2.24 \| 0 \| \| REACT_FORMATION OF THE TERNARY COMPLEX, AND SUBSEQUENTLY, THE 43S COMPLEX \| 39 \| -2.21 \| 0 \| \| REACT_PLATELET DEGRANULATION \| 46 \| -2.19 \| 0 \| \| REACT_CDK-MEDIATED PHOSPHORYLATION AND REMOVAL OF CDC6 \| 45 \| -2.17 \| 0 \| \| REACT_UBIQUITIN-DEPENDENT DEGRADATION OF CYCLIN D1 \| 45 \| -2.15 \| 0 \| \| REACT_UBIQUITIN-DEPENDENT DEGRADATION OF CYCLIN D \| 45 \| -2.13 \| 0 \| \| KEGG_PROTEASOME \| 40 \| -2.13 \| 0 \| \| REACT_DESTABILIZATION OF MRNA BY AUF1 (HNRNP D0) \| 50 \| -2.12 \| 0 \| \| REACT_SCF(SKP2)-MEDIATED DEGRADATION OF P27_P21 \| 49 \| -2.11 \| 0 \| \| REACT_REGULATION OF APOPTOSIS \| 51 \| -2.09 \| 0 \| \| REACT_REGULATION OF ACTIVATED PAK-2P34 BY PROTEASOME MEDIATED DEGRADATION \| 45 \| -2.09 \| 0 \| \| REACT_RESPONSE TO ELEVATED PLATELET CYTOSOLIC CA2+ \| 50 \| -2.07 \| 0 \| \| WIP_HS_PROTEASOME_DEGRADATION \| 56 \| -2.07 \| 0 \| \| REACT_P53-INDEPENDENT DNA DAMAGE RESPONSE \| 45 \| -2.04 \| 0 \| \| REACT_REGULATION OF ORNITHINE DECARBOXYLASE (ODC) \| 44 \| -2.03 \| 0.01 \| \| REACT_AUTODEGRADATION OF CDH1 BY CDH1_APC_C \| 55 \| -2.02 \| 0 \| \| REACT_VIF-MEDIATED DEGRADATION OF APOBEC3G \| 49 \| -2.02 \| 0 \| \| REACT_UBIQUITIN MEDIATED DEGRADATION OF PHOSPHORYLATED CDC25A \| 45 \| -2.02 \| 0 \| \| REACT_APC_C_CDC20 MEDIATED DEGRADATION OF SECURIN \| 55 \| -2.01 \| 0.01 \| \| REACT_P53-INDEPENDENT G1_S DNA DAMAGE CHECKPOINT \| 45 \| -1.99 \| 0.01 \| \| REACT_CDT1 ASSOCIATION WITH THE CDC6_ORC_ORIGIN COMPLEX \| 49 \| -1.98 \| 0.01 \| \| REACT_DNA REPLICATION PRE-INITIATION \| 60 \| -1.97 \| 0.01 \| \| REACT_M_G1 TRANSITION \| 60 \| -1.97 \| 0.01 \| \| REACT_ACTIVATION OF APC_C AND APC_C_CDC20 MEDIATED DEGRADATION OF MITOTIC PROTEINS \| 56 \| -1.97 \| 0.01 \| \| REACT_APC_C_CDC20 MEDIATED DEGRADATION OF MITOTIC PROTEINS \| 56 \| -1.97 \| 0.01 \| \| REACT_CYCLIN E ASSOCIATED EVENTS DURING G1_S TRANSITION \| 54 \| -1.97 \| 0.01 \| \| REACT_SCF-BETA-TRCP MEDIATED DEGRADATION OF EMI1 \| 48 \| -1.96 \| 0.01 \| \| REACT_CDC20_PHOSPHO-APC_C MEDIATED DEGRADATION OF CYCLIN A \| 56 \| -1.95 \| 0.01 \| \| REACT_MITOTIC G1-G1_S PHASES \| 76 \| -1.93 \| 0.01 \| \| REACT_SYNTHESIS OF DNA \| 73 \| -1.92 \| 0.01 \| \| REACT_CYCLIN A_CDK2-ASSOCIATED EVENTS AT S PHASE ENTRY \| 56 \| -1.91 \| 0.01 \| \| REACT_REGULATION OF APC_C ACTIVATORS BETWEEN G1_S AND EARLY ANAPHASE \| 61 \| -1.91 \| 0.01 \| \|  \|  \|  \|  \| \| **Table S4. Downregulated gene sets continued** \|  \|  \|  \| \| KEGG_RIBOSOME \| 66 \| -1.91 \| 0.01 \| \| KEGG_HUNTINGTON'S DISEASE \| 126 \| -1.89 \| 0.01 \| \| REACT_FORMATION OF A POOL OF FREE 40S SUBUNITS \| 74 \| -1.88 \| 0.01 \| \| REACT_ASSEMBLY OF THE PRE-REPLICATIVE COMPLEX \| 53 \| -1.88 \| 0.01 \| \| REACT_REGULATION OF DNA REPLICATION \| 58 \| -1.87 \| 0.01 \| \| REACT_G1_S TRANSITION \| 72 \| -1.87 \| 0.01 \| \| REACT_AUTODEGRADATION OF THE E3 UBIQUITIN LIGASE COP1 \| 47 \| -1.86 \| 0.01 \| \| REACT_VIRAL MRNA TRANSLATION \| 63 \| -1.86 \| 0.01 \| \| REACT_PREFOLDIN MEDIATED TRANSFER OF SUBSTRATE TO CCT_TRIC \| 18 \| -1.86 \| 0.01 \| \| REACT_COOPERATION OF PREFOLDIN AND TRIC_CCT IN ACTIN AND TUBULIN FOLDING \| 18 \| -1.85 \| 0.01 \| \| REACT_INFLUENZA VIRAL RNA TRANSCRIPTION AND REPLICATION \| 63 \| -1.85 \| 0.01 \| \| REACT_SWITCHING OF ORIGINS TO A POST-REPLICATIVE STATE \| 58 \| -1.85 \| 0.01 \| \| BIOC_PROTEASOMEPATHWAY \| 20 \| -1.84 \| 0.01 \| \| REACT_ORC1 REMOVAL FROM CHROMATIN \| 58 \| -1.84 \| 0.01 \| \| REACT_VPU MEDIATED DEGRADATION OF CD4 \| 47 \| -1.84 \| 0.01 \| \| KEGG_ALZHEIMER'S DISEASE \| 112 \| -1.83 \| 0.01 \| \| REACT_RIBOSOMAL SCANNING AND START CODON RECOGNITION \| 44 \| -1.83 \| 0.01 \| \| REACT_EUKARYOTIC TRANSLATION TERMINATION \| 65 \| -1.82 \| 0.02 \| \| REACT_REMOVAL OF LICENSING FACTORS FROM ORIGINS \| 58 \| -1.81 \| 0.02 \| \| REACT_P53-DEPENDENT G1 DNA DAMAGE RESPONSE \| 51 \| -1.81 \| 0.02 \| \| REACT_S PHASE \| 82 \| -1.8 \| 0.02 \| \| REACT_P53-DEPENDENT G1_S DNA DAMAGE CHECKPOINT \| 51 \| -1.8 \| 0.02 \| \| REACT_APC_C-MEDIATED DEGRADATION OF CELL CYCLE PROTEINS \| 63 \| -1.79 \| 0.02 \| \| REACT_TRANSLATION INITIATION COMPLEX FORMATION \| 44 \| -1.78 \| 0.02 \| \| REACT_REGULATION OF MITOTIC CELL CYCLE \| 63 \| -1.77 \| 0.02 \| \| REACT_STABILIZATION OF P53 \| 48 \| -1.77 \| 0.02 \| \| REACT_NONSENSE MEDIATED DECAY INDEPENDENT OF THE EXON JUNCTION COMPLEX \| 70 \| -1.77 \| 0.02 \| \| REACT_G1_S DNA DAMAGE CHECKPOINTS \| 51 \| -1.76 \| 0.02 \| \| REACT_SIGNALING BY WNT \| 60 \| -1.75 \| 0.02 \| \| REACT_EUKARYOTIC TRANSLATION ELONGATION \| 67 \| -1.75 \| 0.02 \| \| REACT_DEGRADATION OF BETA-CATENIN BY THE DESTRUCTION COMPLEX \| 60 \| -1.74 \| 0.02 \| \| REACT_REGULATION OF BETA-CELL DEVELOPMENT \| 67 \| -1.73 \| 0.03 \| \| REACT_INSULIN SYNTHESIS AND PROCESSING \| 101 \| -1.72 \| 0.03 \| \| KEGG_SPLICEOSOME \| 111 \| -1.71 \| 0.03 \| \| REACT_REGULATION OF GENE EXPRESSION IN BETA CELLS \| 67 \| -1.71 \| 0.03 \| \| REACT_POST-ELONGATION PROCESSING OF INTRONLESS PRE-MRNA \| 17 \| -1.7 \| 0.03 \| \| REACT_CHROMOSOME MAINTENANCE \| 47 \| -1.69 \| 0.03 \| \| REACT_NUCLEOSOME ASSEMBLY \| 25 \| -1.69 \| 0.03 \| \| PPARA_TARGETS \| 81 \| -1.68 \| 0.04 \| \| REACT_PROCESSING OF CAPPED INTRONLESS PRE-MRNA \| 17 \| -1.68 \| 0.04 \| \|  \|  \|  \|  \| | | | | |

Size indicates the number of total genes which are involve in the corresponding pathway. (NES): normalised enrichment scores and (FDR): false discovery rate

**Table S5.**Fasting (T0) and postprandial effects of abdominally obese

that underwent the control intervention.

| Metabolites | FC(T0) | iAUC |
| --- | --- | --- |
|  |  | *P* |
| *Acylcarnitines* |  |  |
| 2.Methylbutyroylcarnitine | 1.13 |  |
| Propionylcarnitine | 1.17 |  |
| Isobutyrylcarnitine | 1.22 | 0.04 |
| Stearoylcarnitine |  | 0.01 |
| Octanoylcarnitine |  | 0.04 |
| Decanoylcarnitine |  | 0.04 |
| *Amino acids and related metabolites* |  |  |
| Choline | 1.02 |  |
| Methionine | 1.17 |  |
| Phosphocholine | 1.15 |  |
| Dimethylglycine | 1.21* |  |
| Leucine | 1.06 |  |
| Isoleucine | 1.07 |  |
| Arginine | 1.15* |  |
| Tyrosine | 1.07 | 0.02 |
| Valine | 1.1* | <0.01 * |
| N6.N6.N6.trimethyl.L.lysine | 1.08* |  |
| Ornithine | 1.06 |  |
| Phenylalanine | 1.1 |  |
| 2.aminoadipic.acid | 1.12 | 0.01 |
| Sarcosine |  | 0.01 |
| Dimethylglycine |  | 0.03 |
| Dimethylamine |  | 0.04 |
| *TCA cycle and related metabolites* |  |  |
| Aspartic acid | 1.18 |  |
| Fumaric acid |  | <0.01* |
| Oxylipins |  |  |
| 12.13.DiHOME |  | <0.01 |
| FC: Fold change (T0, T2, before and after control intervention, respectively), iAUC: Incremental area under the curve, *: Significant at *P<0.05,*lFDR<0.2. | | |

**Table S6.** Overview of significantly (FDR<0.2) upregulated and downregulated gene sets in abdominally obese subjects in response to mixed meal challenge before and after weight loss intervention (Δ T4-T0, D2, vs. Δ T4-T0, D1).

**Upregulated gene sets**

| NAME | SIZE | NES | FDR q-val | Direction |
| --- | --- | --- | --- | --- |
|  |  |  |  | D2 D1 |
| WIP_HS_OXIDATIVE_PHOSPHORYLATION | 46 | 2.09 | 0.03 | up down |
| KEGG_OXIDATIVE PHOSPHORYLATION | 92 | 1.94 | 0.09 | up down |
| WIP_HS_ELECTRON_TRANSPORT_CHAIN | 76 | 1.87 | 0.14 | up down |
| REACT_RESPIRATORY ELECTRON TRANSPORT | 56 | 1.84 | 0.14 | up down |
| REACT_RESPIRATORY ELECTRON TRANSPORT | 69 | 1.83 | 0.12 | up down |
| KEGG_PARKINSON'S DISEASE | 86 | 1.77 | 0.18 | up down |
|  | | | | |
| **Downregulated gene sets** | | | | |

| NAME | SIZE | NES | FDR  q-val | Direction |
| --- | --- | --- | --- | --- |
|  |  |  |  | D2 D1 |
| KEGG_GALACTOSE METABOLISM | 17 | -2.26 | 0.00 | down up |
| NCI_HIF1_TFPATHWAY | 44 | -2.07 | 0.03 | down up |
| WIP_HS_ESTROGEN_SIGNALING_PATHWAY | 17 | -2.01 | 0.05 | down up |
| KEGG_CARBOHYDRATE DIGESTION AND ABSORPTION | 19 | -1.92 | 0.06 | down up |
| BIOC_KERATINOCYTEPATHWAY | 35 | -1.93 | 0.07 | down up |
| REACT_NRAGE SIGNALS DEATH THROUGH JNK | 30 | -1.93 | 0.08 | down up |
| WIP_HS_PHYSIOLOGICAL_AND_PATHOLOGICAL_HYPERTROPHY_OF_THE_HEART | 17 | -1.95 | 0.08 | down up |
| NCI_IL6_7PATHWAY | 35 | -1.82 | 0.09 | down up |
| REACT_ION TRANSPORT BY P-TYPE ATPASES | 17 | -1.82 | 0.09 | down up |
| NCI_EPOPATHWAY | 27 | -1.80 | 0.09 | down up |
| WIP_HS_IL-3_SIGNALING_PATHWAY | 37 | -1.88 | 0.09 | down up |
| BIOC_NTHIPATHWAY | 20 | -1.83 | 0.09 | down up |
| WIP_HS_SREBP_SIGNALLING | 22 | -1.85 | 0.09 | down up |
| NCI_ERBB2ERBB3PATHWAY | 31 | -1.80 | 0.09 | down up |
| KEGG_PHOSPHATIDYLINOSITOL SIGNALING SYSTEM | 55 | -1.84 | 0.09 | down up |
| WIP_HS_WNT_SIGNALING_PATHWAY | 28 | -1.83 | 0.10 | down up |
| REACT_ION CHANNEL TRANSPORT | 18 | -1.86 | 0.10 | down up |
| NCI_IL2_1PATHWAY | 46 | -1.81 | 0.10 | down up |
| WIP_HS_RANKL-RANK_SIGNALING_PATHWAY | 41 | -1.86 | 0.10 | down up |
| WIP_HS_KIT_RECEPTOR_SIGNALING_PATHWAY | 39 | -1.77 | 0.12 | down up |
| REACT_GLUCOSE TRANSPORT | 33 | -1.75 | 0.12 | down up |
| REACT_HEXOSE TRANSPORT | 33 | -1.73 | 0.12 | down up |
| NCI_MAPKTRKPATHWAY | 27 | -1.74 | 0.13 | down up |
| **Table S6. Downregulated gene sets continued** |  |  |  |  |
| KEGG_ADIPOCYTOKINE SIGNALING PATHWAY | 40 | -1.75 | 0.13 | down up |
| WIP_HS_MICRORNAS_IN_CARDIOMYOCYTE_HYPERTROPHY | 53 | -1.76 | 0.13 | down up |
| NCI_ATF2_PATHWAY | 27 | -1.75 | 0.13 | down up |
| KEGG_STARCH AND SUCROSE METABOLISM | 20 | -1.72 | 0.13 | down up |
| NCI_AVB3_OPN_PATHWAY | 26 | -1.73 | 0.13 | down up |
| NCI_IL1PATHWAY | 26 | -1.74 | 0.13 | down up |
| NCI_NECTIN_PATHWAY | 19 | -1.71 | 0.13 | down up |
| NCI_AMB2_NEUTROPHILS_PATHWAY | 25 | -1.71 | 0.13 | down up |
| NCI_AR_TF_PATHWAY | 39 | -1.71 | 0.13 | down up |
| KEGG_NEUROTROPHIN SIGNALING PATHWAY | 88 | -1.68 | 0.14 | down up |
| WIP_HS_IL-1_PATHWAY | 42 | -1.68 | 0.14 | down up |
| BIOC_IL1RPATHWAY | 22 | -1.69 | 0.14 | down up |
| KEGG_FOCAL ADHESION | 93 | -1.68 | 0.15 | down up |
| REACT_SIGNALLING TO ERKS | 25 | -1.69 | 0.15 | down up |
| NCI_IL23PATHWAY | 18 | -1.65 | 0.15 | down up |
| REACT_RHO GTPASE CYCLE | 77 | -1.65 | 0.15 | down up |
| REACT_P75 NTR RECEPTOR-MEDIATED SIGNALLING | 62 | -1.65 | 0.15 | down up |
| REACT_SIGNALING BY RHO GTPASES | 77 | -1.65 | 0.15 | down up |
| NCI_ANGIOPOIETINRECEPTOR_PATHWAY | 34 | -1.67 | 0.15 | down up |
| KEGG_ECM-RECEPTOR INTERACTION | 15 | -1.65 | 0.16 | down up |
| NCI_PI3KCIPATHWAY | 41 | -1.66 | 0.16 | down up |
| BIOC_IL7PATHWAY | 15 | -1.64 | 0.16 | down up |
| REACT_INTEGRIN CELL SURFACE INTERACTIONS | 37 | -1.64 | 0.16 | down up |
| BIOC_GLEEVECPATHWAY | 21 | -1.63 | 0.16 | down up |
| WIP_HS_IL-7_SIGNALING_PATHWAY | 22 | -1.65 | 0.16 | down up |
| NCI_VEGFR1_2_PATHWAY | 56 | -1.63 | 0.16 | down up |
| REACT_MITOCHONDRIAL TRNA AMINOACYLATION | 15 | -1.66 | 0.16 | down up |
| WIP_HS_FOCAL_ADHESION | 91 | -1.63 | 0.16 | down up |
| REACT_SIGNALLING BY NGF | 160 | -1.66 | 0.16 | down up |
| BIOC_PPARAPATHWAY | 35 | -1.62 | 0.17 | down up |
| REACT_INTERACTIONS OF THE IMMUNOGLOBULIN SUPERFAMILY (IGSF) MEMBER PROTEINS | 31 | -1.58 | 0.17 | down up |
| KEGG_MISMATCH REPAIR | 18 | -1.59 | 0.17 | down up |
| REACT_NCAM SIGNALING FOR NEURITE OUT-GROWTH | 21 | -1.59 | 0.17 | down up |
| WIP_HS_INTEGRIN-MEDIATED_CELL_ADHESION | 59 | -1.62 | 0.17 | down up |
| REACT_NETRIN-1 SIGNALING | 19 | -1.57 | 0.17 | down up |
| WIP_HS_EGF_RECEPTOR_SIGNALING_PATHWAY | 116 | -1.58 | 0.17 | down up |
| KEGG_FC EPSILON RI SIGNALING PATHWAY | 51 | -1.59 | 0.17 | down up |
| WIP_HS_IL-4_SIGNALING_PATHWAY | 36 | -1.59 | 0.17 | down up |
| NCI_GMCSF_PATHWAY | 31 | -1.59 | 0.17 | down up |
| KEGG_ALDOSTERONE-REGULATED SODIUM REABSORPTION | 17 | -1.58 | 0.17 | down up |
| REACT_REGULATION OF GLUCOKINASE BY GLUCOKINASE REGULATORY PROTEIN | 27 | -1.58 | 0.17 | down up |
| NCI_NETRIN_PATHWAY | 19 | -1.58 | 0.17 | down up |
| NCI_NETRIN_PATHWAY | 19 | -1.58 | 0.17 | down up |
| NCI_ATM_PATHWAY | 27 | -1.59 | 0.17 | down up |
| BIOC_EDG1PATHWAY | 15 | -1.58 | 0.17 | down up |
| **Table S6. Downregulated gene sets continued** |  |  |  |  |
| REACT_GTP HYDROLYSIS AND JOINING OF THE 60S RIBOSOMAL SUBUNIT | 83 | -1.57 | 0.17 | down up |
| REACT_SIGNALING BY INTERLEUKINS | 85 | -1.57 | 0.17 | down up |
| BIOC_TNFR2PATHWAY | 16 | -1.61 | 0.17 | down up |
| KEGG_RIBOSOME BIOGENESIS IN EUKARYOTES | 57 | -1.60 | 0.17 | down up |
| REACT_EFFECTS OF PIP2 HYDROLYSIS | 15 | -1.61 | 0.17 | down up |
| NCI_FAK_PATHWAY | 47 | -1.60 | 0.17 | down up |
| NCI_IFNGPATHWAY | 35 | -1.60 | 0.18 | down up |
| BIOC_INTEGRINPATHWAY | 26 | -1.56 | 0.18 | down up |
| WIP_HS_EBV_LMP1_SIGNALING | 16 | -1.56 | 0.18 | down up |
| NCI_MTOR_4PATHWAY | 58 | -1.60 | 0.18 | down up |
| REACT_SIGNALLING TO RAS | 18 | -1.60 | 0.18 | down up |
| NCI_CXCR4_PATHWAY | 78 | -1.55 | 0.18 | down up |
| KEGG_TIGHT JUNCTION | 61 | -1.54 | 0.18 | down up |
| NCI_CD40_PATHWAY | 23 | -1.55 | 0.18 | down up |
| REACT_GLOBAL GENOMIC NER (GG-NER) | 27 | -1.55 | 0.18 | down up |
| KEGG_INOSITOL PHOSPHATE METABOLISM | 42 | -1.55 | 0.19 | down up |
| KEGG_HOMOLOGOUS RECOMBINATION | 17 | -1.54 | 0.19 | down up |
| BIOC_IL2RBPATHWAY | 28 | -1.54 | 0.19 | down up |
| NCI_REG_GR_PATHWAY | 54 | -1.53 | 0.19 | down up |
| KEGG_VEGF SIGNALING PATHWAY | 45 | -1.53 | 0.19 | down up |
| REACT_NUCLEAR IMPORT OF REV PROTEIN | 30 | -1.53 | 0.19 | down up |
| WIP_HS_INSULIN_SIGNALING | 113 | -1.53 | 0.19 | down up |
| REACT_APOPTOTIC EXECUTION PHASE | 36 | -1.50 | 0.19 | down up |
| NCI_ERBB4_PATHWAY | 20 | -1.50 | 0.20 | down up |

Size indicates the number of total genes which are involve in the corresponding pathway.(NES): normalised enrichment scores and (FDR): false discovery rate

**Table S7.** Overview of downstream oxylipins produced from AA, LA, ALA, DGLA, EPA, and DHA by the COX, LOX and CYP oxidizing enzymes. Spearman correlations between oxylipins and their precursors (AA and LA) have been presented (P<0.05)

| **Precursor** | **Downstream oxylipin** | | **Enzyme** | ***P*** | Ρ |
| --- | --- | --- | --- | --- | --- |
| **AA** | | 5.HETE | 5-LOX | <0.001 | 0.34 |
|  |  | 11.HETE | COX2 | <0.001 | 0.37 |
|  |  | PGE2 | COX | <0.001 | 0.24 |
|  |  | TXB2 | COX | <0.001 | 0.22 |
|  |  | 12S.HHTrE | COX0 | <0.001 | 0.17 |
| **LA** | | 12.13.EpOME | CYP450 | NS |  |
|  |  | 9.HODE | 5-LOX | NS |  |
|  |  | 13.HODE | 15-LOX | <0.001 | 0.30 |
|  |  | 12.13.DiHOME | CYP4500 | 0.003 | -0.14 |
|  |  | 9.12.13.TriHOME | CYP450 | <0.001 | -0.42 |
| **ALA** | | 9.HOTrE | LOX | <0.001 | -0.20 |
| **DGLA** | | PGF1a | COX |  |  |
| **EPA** | | 17.18.DiHETE | CYP450 |  |  |
| **DHA** | | 19.20.DiHDPA | CYP450 |  |  |
|  |  | 10.HDoHE | Auto-oxidation |  |  |

ρ: Spearman rank correlation coefficient, NS: Not significant.

**Table S8.** Amino acid (AAAC) and fatty acid (FAAC) derived acylcarnitines covered in this study.

|  | -Carnitine | Abbreviation | Origin |
| --- | --- | --- | --- |
| AAAC (C_2-5_) | Acetyl- | C_2_ | Leu, Ile |
|  | Proprionyl- | C_3_ | Ile, Val |
|  | Isobutyryl- | C_4_ | Val |
|  | 2-Methylbutyryl- | C_5_ | Ile |
|  | Isovaleryl- | C_5_ | Leu |
| FAAC (C_6-18_) | Hexanoyl- | C_6:0_ | Caproic, hexanoic acid |
|  | Octanoyl- | C_8:0_ | Octanoic acid |
|  | Octenoyl- | C_8:1_ | Octenoic acid |
|  | Decanoyl- | C_10:0_ | Decanoiccapric acid |
|  | Myristoyl- | C_12:0_ | Myristic acid |
|  | Tetradecanoyl | C_14:0_ |  |
|  | Palmitoyl- | C_16:0_ | Palmitic acid |
|  | Stearoyl- | C_18:0_ | Stearic acid |
|  | Linoleyl- | C_18:2_ |  |
|  |  |  |  |

**Table S9.**Correlation of ∆Nadir and Nadir of plasma acylcarnitines levels with phenotypic parameters.

|  | **∆Nadir _vs._ HOMA** | | **∆Nadir _vs._ LBM** | | **Nadir _vs._ HOMA** | | **Nadir _vs._ LBM** | |
| --- | --- | --- | --- | --- | --- | --- | --- | --- |
| **Acylcarnitines** | ***P*** | **ρ** | ***P*** | **ρ** | ***P*** | **ρ** | ***P*** | **ρ** |
| Acetylcarnitine (C_2_) | 0.01 | 0.30 |  |  | 0.03 | 0.26 |  |  |
| 2Methylbutyrilcarnitine (C_4_) | 0.04 | -0.25 |  |  |  |  |  |  |
| Deoxycarnitine (C_0_) | 0.03 | -0.26 |  |  |  |  |  |  |
| Tetradecanoylcarnitine (C_14_) |  |  | 0.005 | 0.34 |  |  |  |  |
| Linoleylcarnitine (C_18:2_) |  |  | 0.02 | 0.37 |  |  |  |  |
| Octanoylcarnitine (C_8_) |  |  | 0.02 | 0.30 |  |  |  |  |
| Tetradecenoylcarnitine (C_14_) |  |  | 0.02 | 0.27 |  |  |  |  |
| Hexanoylcarnitine (C_6_) |  |  |  |  | 0.001 | 0.39 |  |  |
| Butyrylcarnitine (C_4_) |  |  |  |  | 0.0003 | 0.44 |  |  |
| Propionylcarnitine (C_3_) |  |  |  |  | 0.001 | 0.39 |  |  |
| Carnitine (C_0_) |  |  |  |  |  |  | 0.01 | 0.30 |
| Isobutyrylcarnitine (C_4_) |  |  |  |  |  |  | 0.01 | -0.31 |

LBM: Lean body mass, ρ: Spearman's rank correlation coefficient. Only correlations with p<0.05 have been indicated.

**References**

1. Shaham O, Wei R, Wang TJ, Ricciardi C, Lewis GD, Vasan RS, et al. Metabolic profiling of the human response to a glucose challenge reveals distinct axes of insulin sensitivity. Molecular systems biology. 2008;4:214.

2. Pellis L, van Erk MJ, van Ommen B, Bakker GC, Hendriks HF, Cnubben NH, et al. Plasma metabolomics and proteomics profiling after a postprandial challenge reveal subtle diet effects on human metabolic status. Metabolomics : Official journal of the Metabolomic Society. 2012;8(2):347-59.

3. Stroeve JH, van Wietmarschen H, Kremer BH, van Ommen B, Wopereis S. Phenotypic flexibility as a measure of health: the optimal nutritional stress response test. Genes & nutrition. 2015;10(3):459.

4. Saccenti E, Hoefsloot HJ, Smilde A, Westerhuis J, Hendriks MWB. Reflections on univariate and multivariate analysis of metabolomics data. Metabolomics : Official journal of the Metabolomic Society. 2014;10(3):361-74.

5. Shrestha A, Mullner E, Poutanen K, Mykkanen H, Moazzami AA. Metabolic changes in serum metabolome in response to a meal. European journal of nutrition. 2015.

6. Krug S, Kastenmuller G, Stuckler F, Rist MJ, Skurk T, Sailer M, et al. The dynamic range of the human metabolome revealed by challenges. Faseb J. 2012;26(6):2607-19.

7. Ramos-Roman MA, Sweetman L, Valdez MJ, Parks EJ. Postprandial changes in plasma acylcarnitine concentrations as markers of fatty acid flux in overweight and obesity. Metabolism: clinical and experimental. 2012;61(2):202-12.

8. Badoud F, Lam KP, Perreault M, Zulyniak MA, Britz-McKibbin P, Mutch DM. Metabolomics Reveals Metabolically Healthy and Unhealthy Obese Individuals Differ in their Response to a Caloric Challenge. PLoS One. 2015;10(8):e0134613.

9. Matthews DE, Marano MA, Campbell RG. Splanchnic bed utilization of glutamine and glutamic acid in humans. The American journal of physiology. 1993;264(6 Pt 1):E848-54.

10. Adeva MM, Calvino J, Souto G, Donapetry C. Insulin resistance and the metabolism of branched-chain amino acids in humans. Amino acids. 2012;43(1):171-81.

11. Elia M, Livesey G. Effects of ingested steak and infused leucine on forelimb metabolism in man and the fate of the carbon skeletons and amino groups of branched-chain amino acids. Clinical science (London, England : 1979). 1983;64(5):517-26.

12. Podebrad F, Heil M, Reichert S, Mosandl A, Sewell AC, Bohles H. 4,5-dimethyl-3-hydroxy-2[5H]-furanone (sotolone)--the odour of maple syrup urine disease. Journal of inherited metabolic disease. 1999;22(2):107-14.
